# Interaction of the Chromatin Remodeling Protein hINO80 with DNA

**DOI:** 10.1371/journal.pone.0159370

**Published:** 2016-07-18

**Authors:** Shweta Mendiratta, Shipra Bhatia, Shruti Jain, Taniya Kaur, Vani Brahmachari

**Affiliations:** Dr. B. R. Ambedkar Center for Biomedical Research, University of Delhi, Delhi, India; Oxford Brookes University, UNITED KINGDOM

## Abstract

The presence of a highly conserved DNA binding domain in INO80 subfamily predicted that INO80 directly interacts with DNA and we demonstrated its DNA binding activity *in vitro*. Here we report the consensus motif recognized by the DBINO domain identified by SELEX method and demonstrate the specific interaction of INO80 with the consensus motif. We show that INO80 significantly down regulates the reporter gene expression through its binding motif, and the repression is dependent on the presence of INO80 but not YY1 in the cell. The interaction is lost if specific residues within the consensus motif are altered. We identify a large number of potential target sites of INO80 in the human genome through *in silico* analysis that can grouped into three classes; sites that contain the recognition sequence for INO80 and YY1, only YY1 and only INO80. We demonstrate the binding of INO80 to a representative set of sites in HEK cells and the correlated repressive histone modifications around the binding motif. In the light of the role of INO80 in homeotic gene regulation in *Drosophila* as an **E**nhancer of **t**rithorax and **p**olycomb protein (ETP) that can modify the effect of both repressive complexes like polycomb as well as the activating complex like trithorax, it remains to be seen if INO80 can act as a recruiter of chromatin modifying complexes.

## Introduction

The SNF2 family of chromatin remodelers is known to facilitate different regulatory functions involving chromatin. The INO80, a highly conserved member of the SNF2 family of DNA dependent ATPase shows functional diversity and is implicated in transcription, replication, cell division and DNA repair [1]. The alteration of chromatin structure to facilitate the recruitment of various interacting complexes for gene expression regulation is brought about by chromatin remodeling and histone modifications to maintain the status of gene expression [2]. One of the mechanisms by which chromatin structure can be altered involves the disruption, mobilization and stabilization of the histone octamer by multiprotein complexes, leading to either repression or activation of transcription [3]. The chromatin remodeling complexes hydrolyze ATP through a subunit that belongs to the superfamily of SNF2-type ATPases which can be considered the catalytic core or engine of the complex [4]. The SNF2 chromatin remodeling factors are divided into distinct subfamilies based on the sequence of their common SNF2 helicase related domains [5]. Apart from the helicase like domain, additional domains characterize different subfamilies of SNF2. For instance, SNF2 subfamily with the same name as the superfamily contains bromodomain; the CHD ATPases (chromodomain helicase DNA-binding) have double chromodomains; the Mi2/NURD subfamily contain two copies of the PHD domain in addition to the chromodomain; the ISWI family has a SANT domain and the INO80 subfamily contains DBINO, a DNA-binding domain [6–8]. So far there has been only a few reports of members of SNF2 family having DNA binding domain; INO80 [7,8], ISWI and CHD1 ([1, 9]. Based on the structural similarity of CHD1 with the SANT and the SLIDE domains of ISWI, the DNA binding domain of CHD1 was identified. By deletion analysis it has been shown that the DNA or the chromatin binding domain of CHD1 is at the C-terminal and the loss of about 450 amino acid residues of the C-terminal including the DNA binding domain leads to the loss of chromatin binding as well as ATPase activity. The minimum DNA binding domain of CHD1 was identified as 265 amino acid stretch (1009–1274 in the C-terminal region). It is speculated that these non-enzymatic motifs function as modules that specifically target the remodeling factors to selected chromatin regions and possibly also have a mechanistic role in nucleosome remodeling [9].

Although some of the remodeling factors demonstrate *in vitro* DNA binding activity to various degrees, specific targeting and recruitment to nucleosomal DNA, as well as regulation of chromatin remodeling activity is believed to be mediated either by specific interactions between specialized domains of chromatin remodeling complexes with post-translationally modified histones or through the interaction with sequence-specific DNA binding proteins, such as YY1, which recruit different complexes including the INO80 complex [10,11]. On the other hand post-translational modification of histones is used as a signal for recruitment by chromodomains of Mi2 subfamily that preferentially interact with di- and trimethylated histone H3K4 [12]. The mechanistic role of non-enzymatic domains is illustrated by ACF1 which contains bromodomain and is essential for nucleosome sliding catalyzed by ISWI [13–17].

The human INO80 is functionally very versatile and is involved in replication, chromosome segregation, DNA repair and replication stress recovery [18–21]. The C-terminal of the INO80 complex is phosphorylated in DNA damage tolerance pathway [22]. The recent work by Wang et al, have shown the requirement of INO80 complex for embryonic stem cell (ESC) self-renewal, activation of pluripotency genes, reprogramming and blastocyst formation in mouse ESC in culture [23]. The INO80 complex in metazoans is highly similar to that of *Sacchromyces cerevisiae* [11]. Both in flies and mammalian cells the targeting of the INO80 complex to genomic sites is by the sequence-specific DNA-binding member of the complex i.e. PHO/YY1 as inferred by the co-existence of the two proteins in the same complex [11,24,25].

Earlier reports from our laboratory led to the identification of the INO80 subfamily as a novel subfamily of the SNF2 group of chromatin remodeling proteins [7]. One of the unique features identified for the INO80 subfamily is the presence of a highly conserved DNA binding domain (DBINO). The presence of DBINO domain (IPR020838) and the conservation of this domain in more than 700 proteins, indicates its functional relevance (http://www.ebi.ac.uk/interpro). We have demonstrated the role of INO80 in homeotic gene regulation by creating functional knockouts of INO80 in *Drosophila melanogaster* [26]. Neumann et al [27] isolated a point mutant and a deletion mutant of dIno80 and observed the lethality in later development stages. However, we have mapped our deletion and narrowed it to the exon 12, further we detect the complete absence of Ino80 transcript (unpublished data) and protein in the null embryos unlike the mutant in the other report [28]. We have shown genetic interaction of INO80 with different polycomb and trithorax genes in *Drosophila* and also the interaction of dIno80 with upstream sequences of homeotic genes in *Drosophila*. The presence of a highly conserved DNA-binding domain (DBINO) within the catalytic subunit of INO80 raises the possibility of its direct interaction with DNA.

In the present manuscript we describe the identification of consensus binding motif for DBINO domain of INO80 using the **S**ystematic **E**volution of **L**igands by **EX**ponential enrichment (SELEX) approach Hyde-DeRuyscher [29]. We demonstrate the direct binding of INO80 with the consensus DNA sequence *in vitro*. We report the transcriptional regulation of reporter genes through the DBINO domain of INO80 which is sensitive to the presence of INO80 siRNA. We demonstrate the *in vivo* interaction of INO80 with regions predicted as its targets based on the presence of the DNA binding sequence motif. Our results point to the possibility of INO80 impacting the interaction of transcription regulatory complexes with the genome.

## Materials and Methods

### Expression and purification of the DBINO domain of INO80

The DBINO domain of human homolog of INO80 (GenBank Accession No. NM_017553, 1050-1428bp) was expression cloned as a GST-fusion protein in pGEX-3T and transformed in *E*.*coli* BL21 as described in Bakshi et al [8]. The reading frame was confirmed by sequencing and the protein was purified using Glutathione sepharose following induction with IPTG.

### Polyclonal Anti-INO80 antibody

The fractions containing purified protein GST-DBINO were pooled and GST tag was cleaved by Factor Xa digestion (2U Factor Xa per 500ng purified protein, for 2hrs at 4°C). The GST tag and Factor Xa were removed from the preparation by passing through GST sepharose and Factor Xa removal resin respectively. The purified DBINO protein was used as an antigen for raising polyclonal anti-INO80 antibody. Western blot was performed using 1:100 dilution of the immune sera (Anti-INO80 antibody) and HRP conjugated anti-rabbit IgG as secondary antibody (1:5000) dilution. The blot was developed using TMB/H2O2 or DAB system. In addition to this anti-INO80 antibody, we have used the commercial anti-INO80 antibody (ab118787) from Abcam plc.

SELEX method: The selection of DBINO binding sites from a pool of random oligonucleotides was performed as described by Hyde-DeRuyscher [29]. The DBINO domain of hINO80 was expression cloned as a GST-fusion protein in pGEX-3T (Amersham) as described earlier [8]. A library of 56 base pair oligonucleotides, contained a central 20 nucleotide random sequence flanked by defined sequences for the binding of PCR primers: 5’CATGAATTCTCCTATACT (N)_20_ TGTATCGATGA ATTCCAC3’. The oligos were captured based on their ability to bind to GST-DBINO immobilized on glutathione sepharose beads. The capture cycle was reiterated six times to minimize the non-specific interaction. The 56-nucleotide random DNA library was PCR amplified using 10pmol of the two primers, 5'CATGAATTCTCCTATACT3' (forward primer) and 5' GTGGAATTCATCGATACA 3' (reverse primer), 10 ng 56mer library, lX PCR buffer (10 mM Tris-HCl pH 8.3, 50 mM KC1, 1.5 mM MgCl2), 0.2 mM of each deoxynucleoside triphosphate and 0.5U Taq DNA polymerase. Amplification (94°C, 20s; 51°C, 20s; 72°C, 20s) was carried out for 25 cycles in a volume of 50μl and subsequently purified using nucleotide purification kit. The final enriched, amplified and purified sequences were ligated to pGEMTeasy vector and cloned in *E*.*coli* DH5α cells. The clones were selected for the presence of insert by PCR with standard M13 primers as well as by restriction digestion with NotI and confirmed by sequencing. The strategy is shown in S2 Fig. The motif shared between the selected oligos was predicted by using MEME program (http://meme.sdsc.edu/meme/website/meme-download.html) [30].

### Mammalian Cell culture

HEK293T cells were propagated in DMEM high glucose media (Gibco, Invitrogen) supplemented with 10% FBS and 1X antibiotic and anti-mycotic solution at 37°C in presence of 5% CO_2_.

### Preparation of nuclear extract

The nuclear extract was prepared as described earlier [31]. About 10^7^ cells were homogenized in cold buffer A (10mM HEPES-KOH pH 7.9, 1.5mM MgCl_2_, 10mM KCl, 0.5mM DTT and 0.2mM PMSF), centrifuged and the supernatant was stored as the cytoplasmic fraction. The pellet was extracted with cold buffer B (20mM HEPES-KOH pH 7.9, 25% glycerol, 420mM NaCl, 1.5mM MgCl_2_, 0.2mM EDTA, 0.5mM DTT and 0.2mM PMSF) and was collected as the nuclear extract and stored in aliquots at -20°C.

### Electrophoretic mobility shift assay (EMSA)

EMSA assays were carried out either with γ^32^P-labeled double stranded oligos bearing single INO80 binding motif or 5’ Cy3 labeled double stranded oligonucleotides having three INO80 binding motifs. The unlabeled oligonucleotides with or without the INO80 binding motif and oligos with site directed alteration were used in different competitive EMSA (S1 Table). The cloned GST-DBINO domain was tested for binding with γ^32^P-labeled double stranded oligos.

The nuclear extract was incubated with 10pmoles of Cy3 labeled double-stranded oligonucleotides, for 20 minutes at room temperature in 1X binding buffer (250mM HEPES, 10mM KCl, 1mM EDTA, 10mM DTT, 1mM PMSF and 100mM NaCl), 1ug Poly (dI-dC), 1ug tRNA and 10% glycerol and was analyzed on 5% acrylamide-bisacrylamide (29:1) gel in 0.5X Tris-borate-EDTA (TBE) buffer or in buffer containing 89mM Tris, 89mM boric acid (pH 7.5), and 2mM MgCl_2_ at 10V/cm at 6°C. The gel was scanned on Typhoon phosphoimager (Typhoon FLA TRIO, GE Biosciences).

For the competition experiments for analyzing the specificity of the interaction, unlabeled specific and non-specific oligos in different ratios were added. The EMSA with nuclear extract with depleted INO80 protein was carried out following transfection of HEK293T cells with siRNA-INO80 (Sigma-EHU069661) or control siRNA (sc-37007). Transfection was carried out at a concentration of 2nmoles od siRNA per T-75 flask for 72 hours. The probe to protein ratio was 1:20 (molar ratio) and was incubated for 2 hours at 37°C. The supershift experiments were performed in 1:5 (probe: protein) molar ratio. The labeled oligos and the nuclear extract were incubated together with hINO80 antibody at 4°C overnight. The reaction mixture was checked on 5% PAGE as described above.

### Reporter constructs and assays

The sequence containing the INO80 binding motif was cloned upstream of the pGL3 promoter with firefly luciferase as the reporter gene using Kpn1 and Xho1 restriction sites to generate BS-UP pGL3 constructs. Similarly, BS-Dn pGL3 was generated by cloning the INO80 binding motif downstream to the poly(A) signal of the reporter gene. For reporter assay, HEK293T cells were transfected with 200ng of control/BS-UP pGL3 or BS-Dn pGL3 and 2ng of pRL Renilla luciferase vector (Promega) as transfection control using Lipofectamine 2000 (Invitrogen). The activity of both firefly and renilla luciferase was measured in cell-free extracts with luminol using the Dual luciferase assay kit (E1910, Promega). The firefly luciferase counts were normalized to that of renilla luciferase and the fold change was calculated with respect to the empty vector. This was performed in five biological replicates each in triplicate. The test of significance used is Student's t-test (two-tailed).

### *In silico* prediction and gene ontology classification

To analyze the presence of the invariant core of the putative INO80 binding motif (5’GTCAGCC 3’) and the YY1 binding motif (5’GCCATCAT3’) in the human genome, the data set consisting of 2000bp sequences upstream of transcription start site of 22,810 genes from human genome (GRCh37.p13) was retrieved. The gene ontology classification of the putative target genes was performed using Panther classification tool that classifies proteins and their genes based on molecular and biological function.

### Chromatin immunoprecipitation and real time PCR

The following antibodies from Abcam plc, were used in the study: anti-INO80 (ab118787), rabbit anti-IgG control (ab46540), anti-H3K27me3 (ab6002-100) and anti-H3K9ac (ab4441). HEK293T cells were used in chromatin immunoprecipitation (ChIP) assay with Anti-INO80 antibody [32].

The ChIP DNA was amplified by PCR: 94°C 5’; 94°C 30”, reannealing for 30”, 72°C for 30” (35 cycles) followed by 72°C 5’. The reannealing temperature for different primers is given in S2A Table. The primers used for Quantitative PCR (qPCR) in partial tiling assays of ChIP with antibody directed against modified histones are given in S2B Table. qPCR was performed to assess the enrichment of putative target genes in ChIP experiments using SYBR green chemistry on ABI SDS7300 platform. The same protocol was followed for the ChIP with antibody for modified histones. The Student's t-test (two-tailed) was used to test the significant interaction relative to that of IgG.

## Results

### Identification of DBINO binding motif

To identify the sequence for interaction, the DBINO domain of hINO80 was cloned and sequenced. The expression of GST-DBINO in the clones was confirmed by immunostaining with anti-GST and anti-DBINO antibody after purification of the protein and comparison of uninduced and induced (with IPTG) clones (S1 Fig). The absence of signal in the extract from un-induced clones indicates the specificity of the antibody (S1A and S1B Fig). The INO80 protein in nuclear extracts from HEK293T cells were detected by using anti-INO80 antibody from AbCam (S1C Fig).

**Fig 1:**
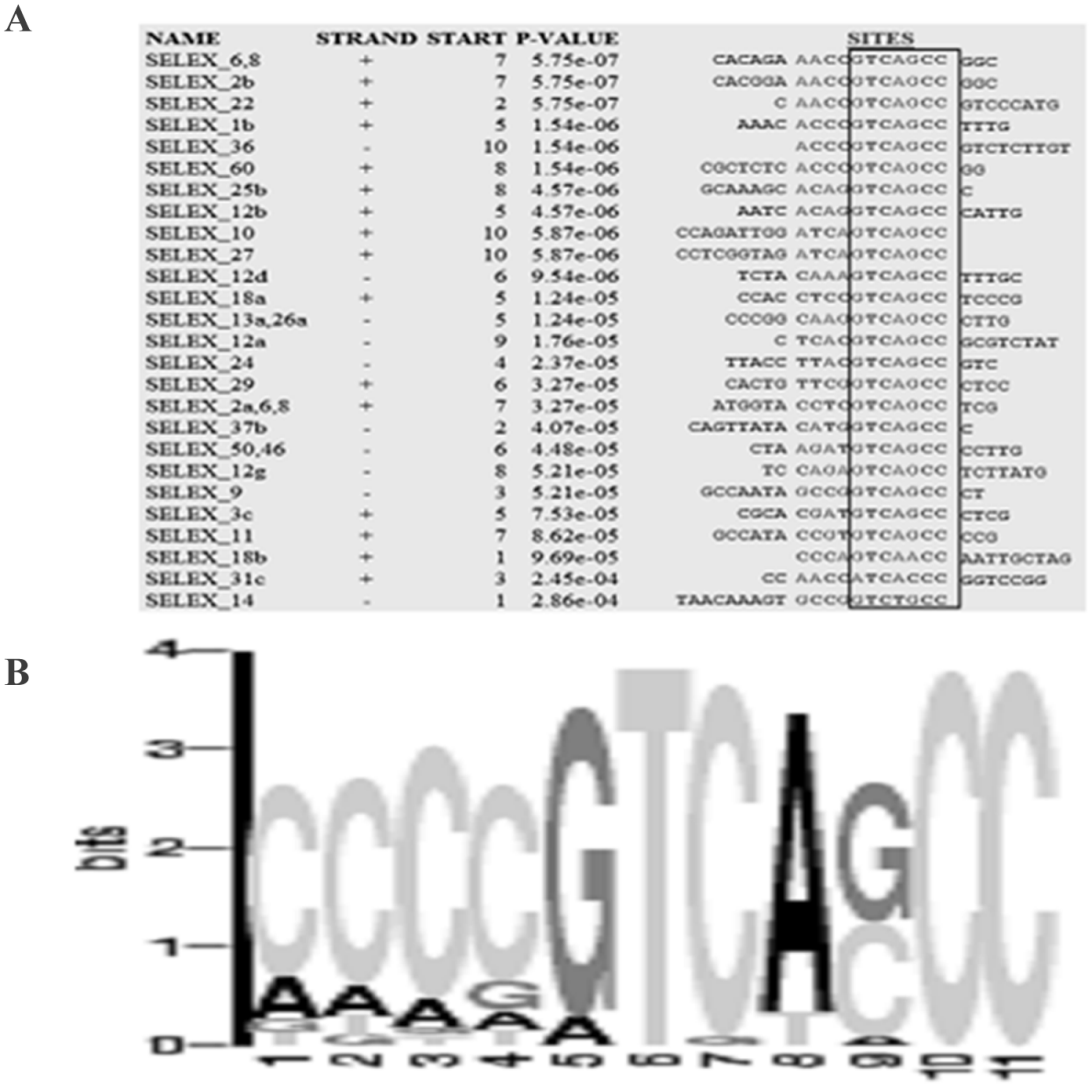
MEME-based prediction of INO80 binding motif. (A) Snapshot of MEME output for the sequences enriched after DBINO interaction, (B) Position weight matrix of the putative INO80 motif generated by MEME.

We identified the DNA binding motif of INO80 using SELEX approach using GST-DBINO immobilized on glutathione sepharose (S2 Fig). After six reiterations of the capture cycle, a pool of 56mer PCR amplicons were cloned and 60 clones were sequenced. Out of the 60 clones, 28 had a single copy of the oligo, while the rest had multiple copies of the 56mer oligonucleotide integrated in tandem. No oligonucleotides were captured on beads with bound GST alone, used as the control. The 59 unique sequences obtained from the SELEX clones (S3 Table) were analyzed for motif enrichment using MEME program. The consensus motif predicted by MEME for INO80 binding is 5’[CA][CA][CA][CG] GTCA[GC]CC 3’ with a significant enrichment score of 5.75e-07 (Fig 1). Thus, the 11 mer consensus motif was identified as the hINO80 binding DNA sequence motif and used in further experiments to study its interaction with the INO80 protein.

### Interaction of INO80 with its putative binding motif

**Fig 2:**
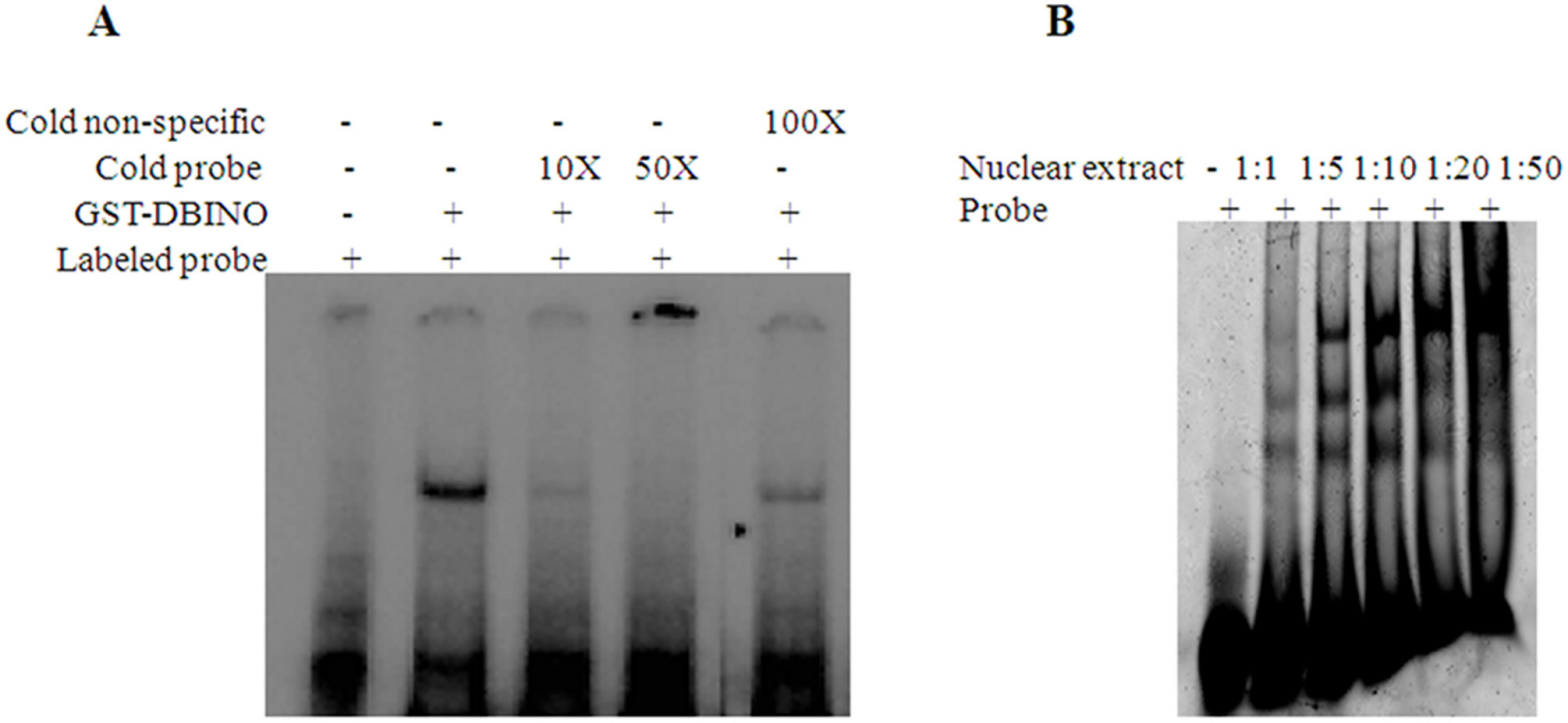
INO80 interaction with SELEX motif. (A) Interaction of GST-DBINO with DNA. (B) Optimization of molar ratio of oligonucleotide: nuclear extract from HEK293T cells for EMSA.

**Fig 3:**
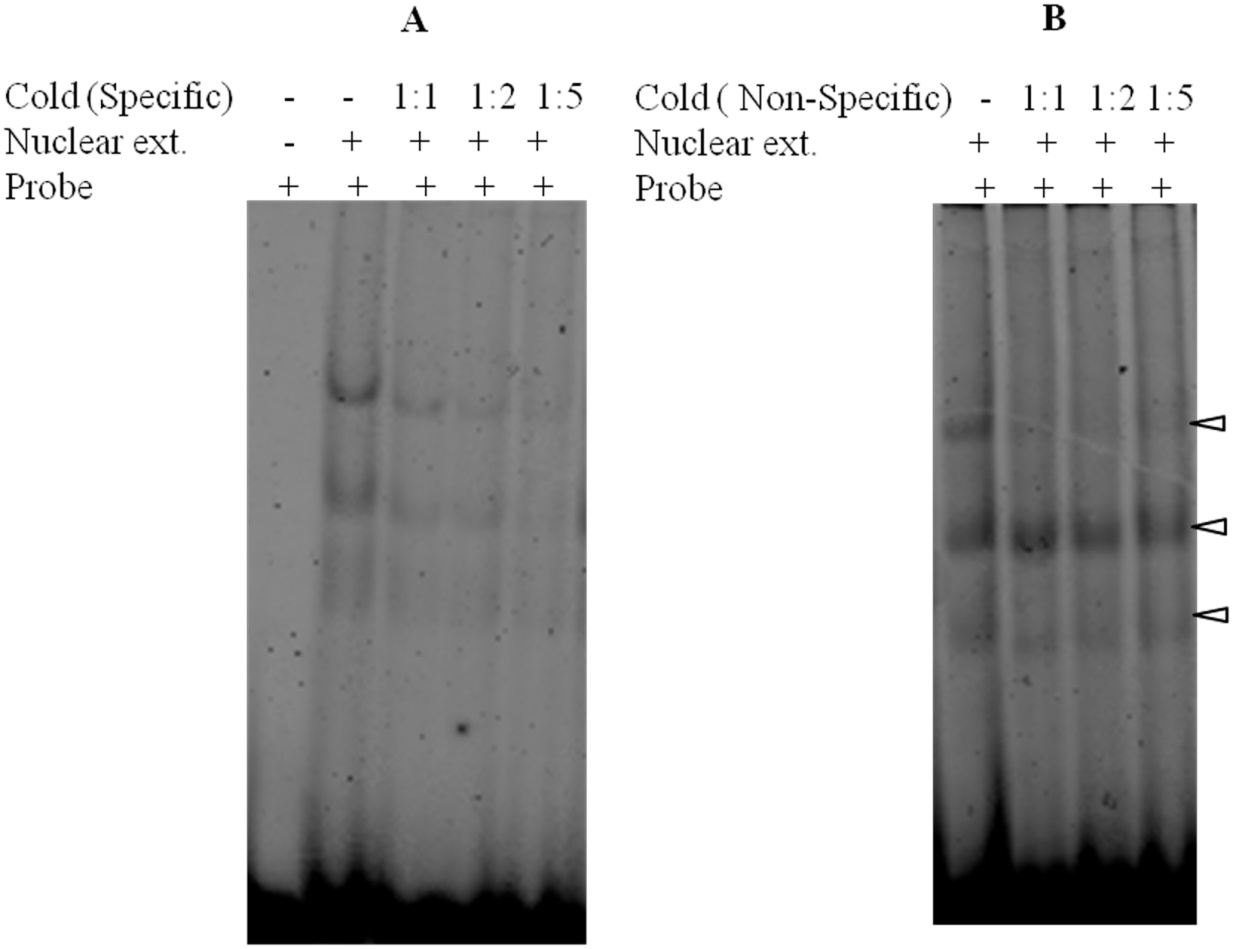
Interaction of INO80 binding motif with nuclear extract from HEK 293T cells. Results of EMSA with Cy3 labeled specific oligo at ratio of 1:5 in all the experiments. The unlabeled competing oligos both specific(A) and non-specific(B) were varied at different molar ratios as indicated at the top of the lanes. The arrow heads indicate the three retarded oligo nucleotides.

**Fig 4:**
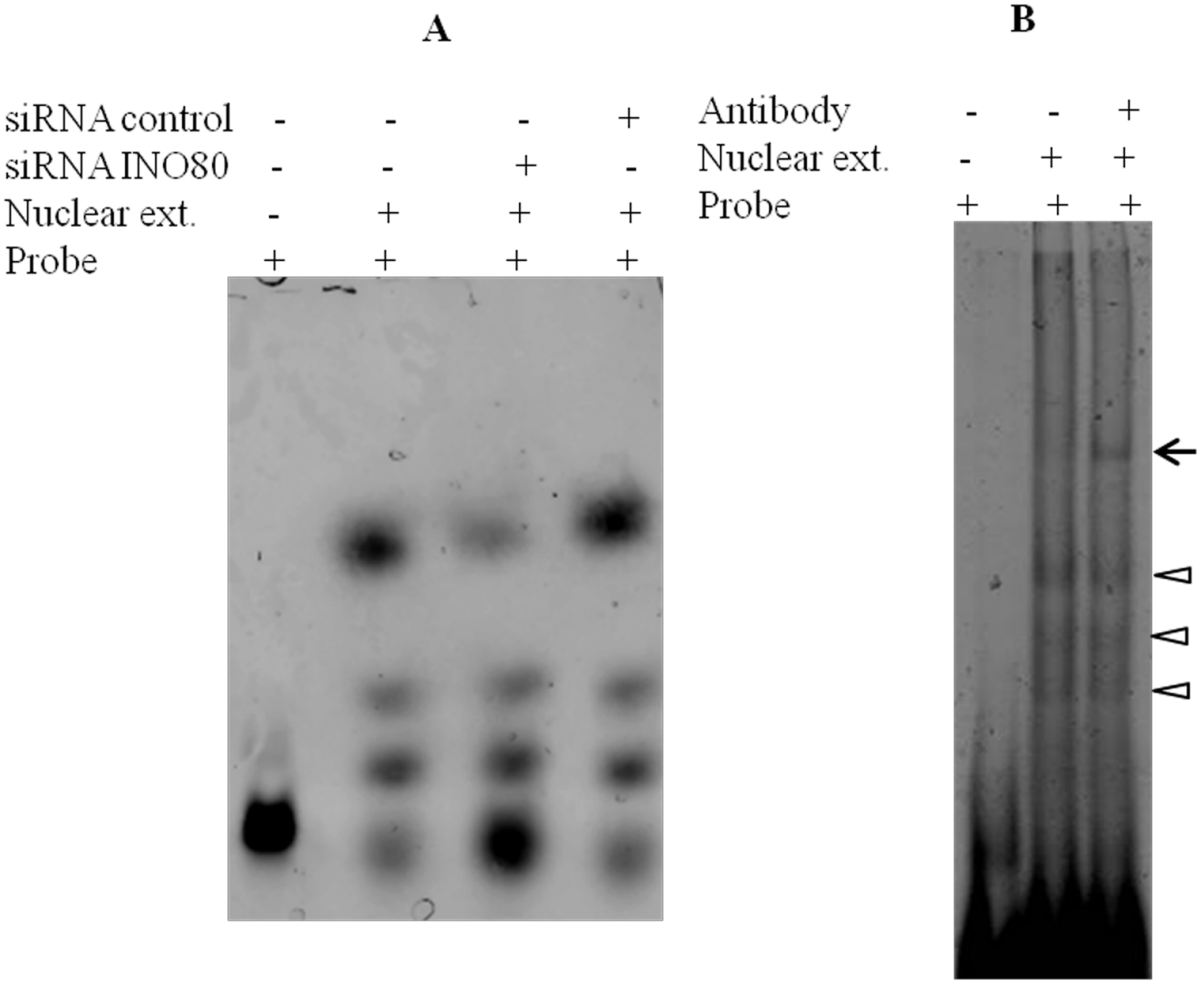
Specificity of interaction of INO80. (A) Interaction of INO80 binding motif with the nuclear extracts from siRNA-INO80 transfected cells (Sigma-EHU069661) compared to untransfected and control siRNA (sc-37007) transfected cells. (B) The supershift of the INO80 bound oligo following the addition of anti-INO80 antibody. The arrow heads indicate the three retarded oligo nucleotides, while the arrow points to the heavier complex formed after addition of the antibody.

We examined the direct interaction of DBINO domain with the predicted consensus motif by EMSA using cloned DBINO domain (Fig 2A) and also nuclear extracts from HEK cells (Fig 2B). A double-stranded oligonucleotide having a single copy of the consensus sequence was incubated with recombinant DBINO. The interaction of the native INO80 with the consensus motif was analysed using the nuclear extract from HEK293T cells and Cy-3 labeled double-stranded oligonucleotides having three tandem repeats of the consensus DNA sequence motif (Fig 2B). The molar ratio of the oligo and the protein in the nuclear extract was varied from 1:1 to 1:50 (oligo: Protein molar ratio) to optimize the binding. The binding is detected at a molar ratio of 1:1 onwards and there is a concentration dependent increase in the intensity of the bound oligo as well as the extent of shift with an increase in protein concentration. The specificity of the binding was examined by unlabeled-specific and unlabeled-non-specific oligonucleotides (Fig 3). We used a ratio of 1:5 as the optimum ratio of DNA: protein for competitive EMSA (Fig 3A and 3B). We observe that the three different retarded bands are competed out starting at 1:1 molar ratio of Cy3-oligo to unlabelled specific oligo, almost complete displacement is seen at 1:5 molar ratio. To demonstrate the involvement of INO80 in this binding, we compared the binding of nuclear extract following knock-down of INO80 in HEK cells (Fig 4A). It is clearly seen that the binding decreases by about 25% as estimated by densitometric analysis. In order to confirm the presence of INO80 in these complexes, super-shift assay was carried out with anti-INO80 antibody (ab118787: Fig 4B). A further retarded band was observed on addition of anti-INO80 antibody confirming the involvement of INO80 in this interaction. These results show that there is a sequence dependent interaction of INO80 with DNA. The interaction of the recombinant DBINO domain appears to be weak, by the ratio of DNA: protein required for binding being high, while the INO80 protein from the nuclear extract exhibits stronger interaction. *In vivo*, the interactions would be mediated by full length INO80 protein and also a complex.

### The specificity of INO80 for bases within the consensus sequence motif

**Fig 5:**
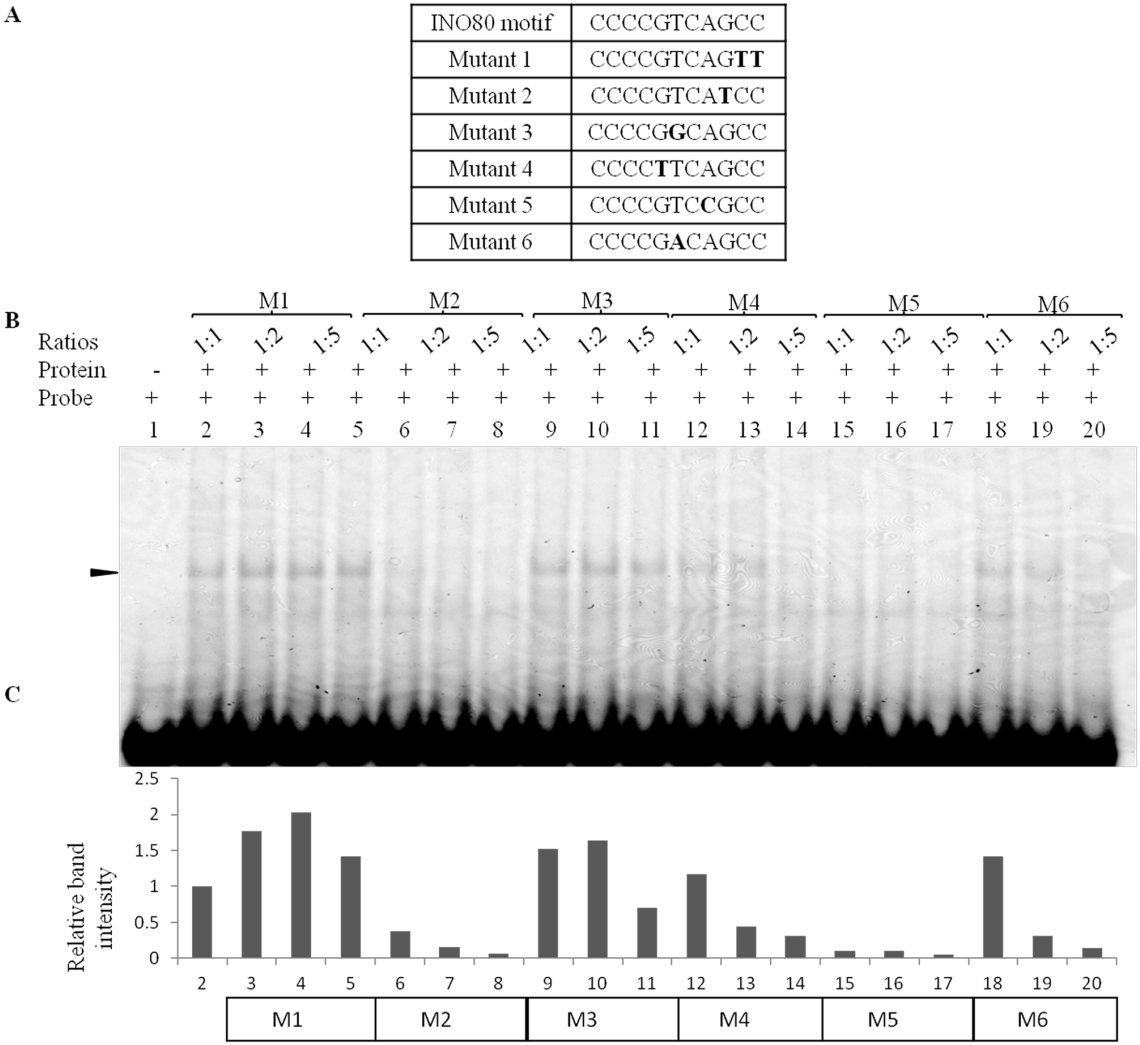
Competitive EMSA with the mutant oligos. (A) List of mutant oligos used in the assays, (B) Results of competitive EMSA with mutant oligos at the indicated ratio, (C) Densitometric scan of gel shown in B. The intensity value of the band (shown by arrow) in the lanes shown in (B), normalized to band at the same position in lane 2, are plotted on the Y-axis. Three different ratios for each competing oligo were used as indicated in B and the mutant sequence used is mentioned below the histogram.

To assess the base specificity within the consensus DNA sequence, the consensus motif was altered at various positions and these oligos were used in the competitive EMSA (Fig 5A). The mutant oligos M1 and M3 were unable to compete with the specific oligo for binding with INO80, while the other four mutant sequences, M2, M4, M5 and M6 were able to compete out the binding of the specific oligo at higher concentrations (1:5; Fig 5B and 5C). The bases important for binding of INO80 within the 11mer sequence from the 5’end are, T at 6^th^ position and the two C residues at position 10 and 11 (CCCCG**T**CAG**CC**). When the T at 6^th^ position is altered to G (M3) there is no interaction, while it is altered to A (M6), the interaction persists at equal molar ratio of 1:1, and is competed out at 1:5, suggesting a decrease in affinity for the mutant oligo. These residues are also among the most conserved residues in the consensus motif. Thus, we confirm the binding of INO80 to its sequence motif and that the binding is specific as seen by competitive EMSA and the INO80 knock-down experiments.

### INO80 regulates gene expression through its DNA binding motif

**Fig 6:**
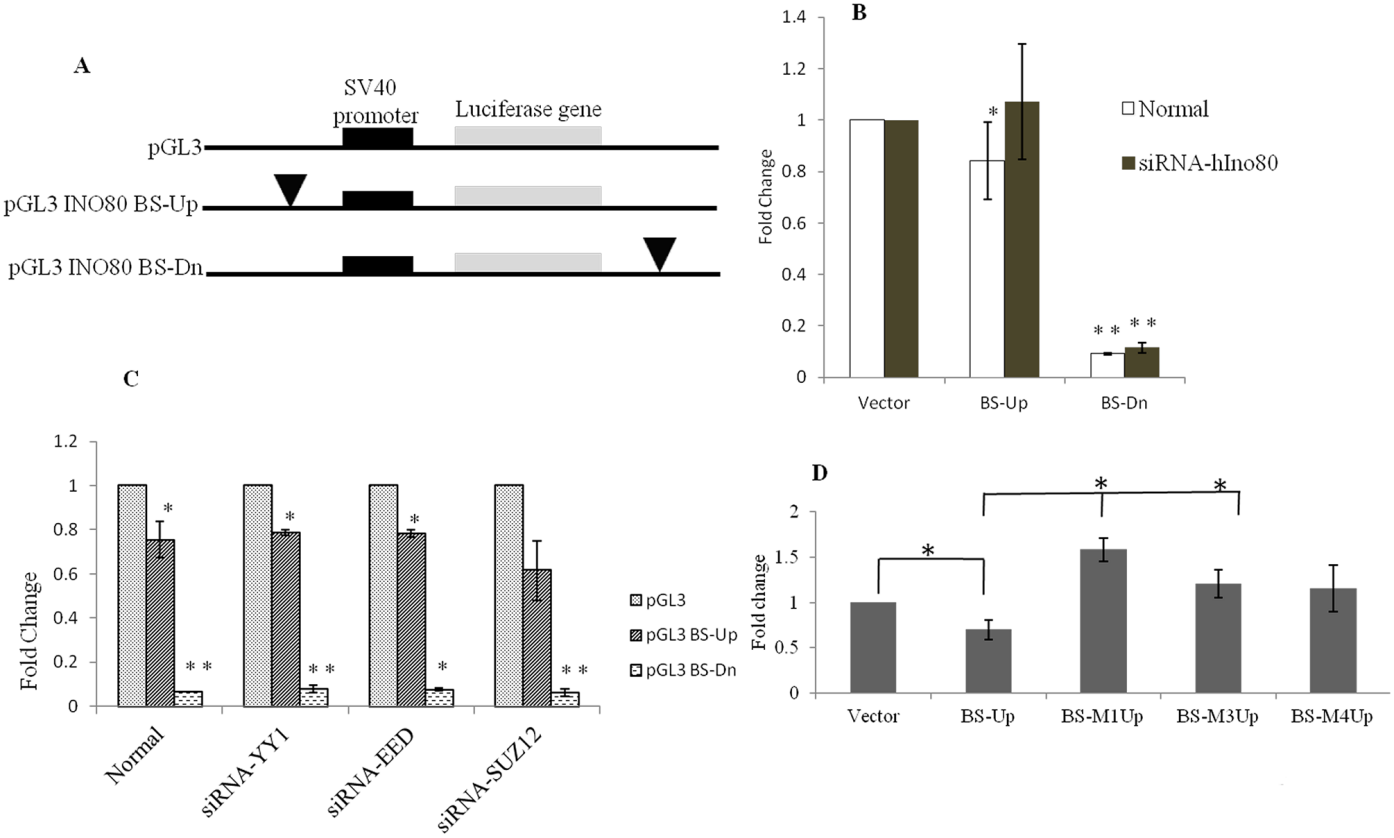
Effect of INO80 binding on expression of luciferase reporter gene in HEK293T cell line. (A) Diagrammatic representation of the constructs used in the luciferase reporter assays. The constructs are; pGL3 promoter vector without the INO80 binding site, pGL3INO80 BS-Up (BS-Up) where the INO80 binding motif is cloned upstream of the promoter of luciferase reporter gene and pGL3INO80 BS-Dn (BS-Dn) where binding motif is cloned down-stream of the poly(A) signal. (B) The effect of INO80 binding on the expression of reporter gene under normal and knock-down of hINO80 condition in HEK293T cells. The reporter expression is indicated as fold change with reference to expression from pGL3 vector. The firefly luciferase counts were normalized with renilla luciferase counts. (C) The effect of knock-down of PRC members on reporter expression. The constructs pGL3, pGL3 BS-up and pGL3 BS-Dn were transfected in cells treated with siRNA-YY1, siRNA-EED and siRNA-SUZ12. (D) The effect of the mutant oligos (Fig 5A) on reporter expression. The error bars represents the standard deviation (*p value<0.05 and **p<0.001).

To examine if INO80 has any regulatory role through its interaction with the consensus sequence of recognition, we cloned the consensus binding sequence upstream of the SV40 promoter in pGL3 vector as well as downstream to the poly(A) signal of the firefly luciferase as the reporter (Fig 6A). It is known that PRC/Trx complexes can impact expression of developmental genes by binding to either the upstream or the downstream regions of the target gene [33]. Since we observe that INO80 interacts with both PcG (Polycomb) and TrxG (trithorax group) proteins in *Drosophila* [28], we have investigated the effect of interaction of INO80 downstream to the reporter gene also.

On transfection of HEK293T cells, we observe a marginal but significant down-regulation of luciferase expression (p<0.05; Fig 6B). As mentioned under methods, this was performed in five biological replicates each in triplicates. The down-regulation is higher in the downstream clone, BS-Dn pGL3 (90.8%). To test the dependence of this negative regulation on INO80 protein, we knocked down INO80 with siRNA and found that the repression of the firefly luciferase activity is lost in BS-Up pGL3 and the activity is regained to the level comparable to that from the control vector (Fig 6B). However in BS-Dn pGL3, there is no effect and the reporter gene continues to be repressed. We tested the involvement of YY1, which is known to recruit INO80 to the chromatin, as well as other PRC members, by knock-down of these proteins using specific siRNA (Fig 6C). The knock-down of YY1, EED and SUZ12 shows no effect on the downregulation of the reporter gene in both the reporter constructs, BS-Up pGL3 and BS-Dn pGL3 (Fig 6C). We have not pursued the repression by the motif cloned downstream further at this point. The specificity of the INO80 mediated effect observed in BS-Up pGL3 is shown by the lack of such an effect in case of BS-Down pGL3. The level INO80 knock down is shown in S3 Fig.

We investigated the ability of the mutant sequences to regulate reporter gene by cloning various mutant oligos in the upstream region in pGL3 vector. The constructs namely; BS-M1Up, BS-M3Up and BS-M4Up were transfected into HEK293T cell line and assayed for luciferase after 24hours and found that the mutant sequences did not bring about any repression and the level of expression was comparable to that of the empty vector while the consensus sequence shows significant difference in activity compared to the mutant sequences (Fig 6D). For example in M1 & M3 the expression was comparable to that of the vector, while in M4 there is no significant loss of repression. This has a correlation to the binding ability of the mutant sequences to INO80 *in vitro*, shown in Fig 5. Thus, implying that the binding of INO80 to its consensus sequence is essential for its regulatory function.

### *In silico* prediction of genes potentially regulated by INO80 in the human genome

We analysed the distribution of the DBINO binding motif (5’GTCAGCC’) in the upstream regions of the annotated protein coding genes in the human genome. The occurrence of the motif in 2000 base pairs upstream of the Transcription Start Site (TSS) for 22,810 genes from the human genome was considered (GRCh37.p13; S4 Fig, S4 Table). The occurrence of the invariant core sequence of the INO80 motif (7mer) and the YY1 motif (8 mer) were detected in the promoter regions of 4696 and 1300 genes respectively. We analyzed the occurrence of a random sequence of similar length as a control, which was significantly low (1126) compared to that of INO80 binding motif (S4 Table). Further, the upstream of certain genes contain INO80 binding sequence motif without YY1 binding site suggesting the possibility of YY1-independent interaction of INO80 with DNA. To analyze the distribution of functional categories of the genes predicted as targets of INO80, we classified putative target genes using PANTHER version 8.1 [34]. At the level of biological functions metabolic process and cellular process were the major class represented and under molecular function binding and catalytic activity were over represented (data not shown). We analyzed the presence of INO80 binding motif in genes known to be targets of PRC1 and PRC2 complexes (S5 Table). Based on this, we analyzed the interaction of INO80 protein *in vivo* in a representative set of genes.

### Interaction of INO80 with target genes *in vivo*

**Fig 7:**
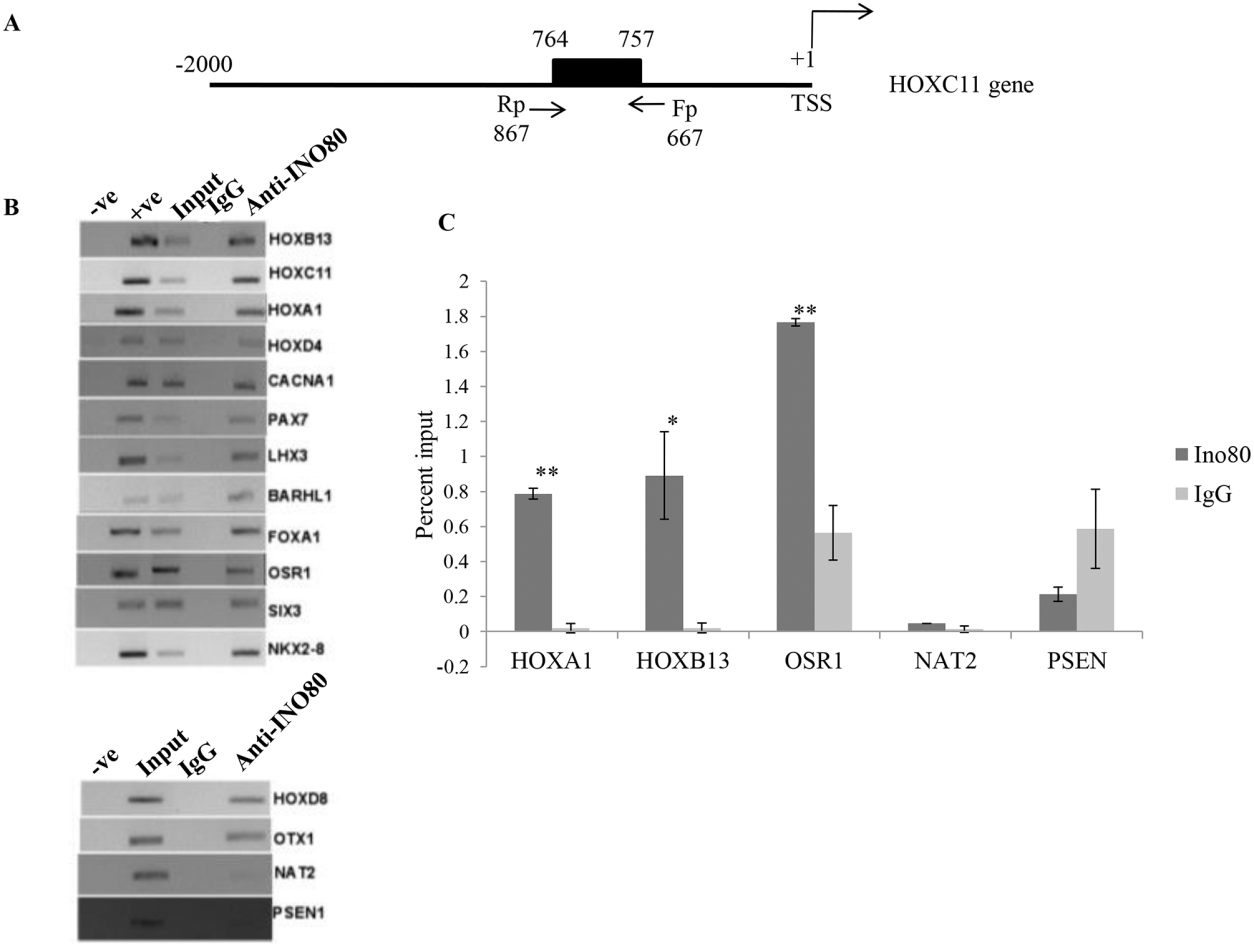
*In vivo* interaction of INO80 on predicted gene targets. (A) A line diagram for the region 2Kb upstream of HOXC11 gene is shown as the example of the region analyzed by ChIP. The filled rectangle denotes the INO80 binding motif and the position of the primers (arrows) are marked. (B) ChIP-PCR results for the indicated genes on the human genome. The interaction of INO80 protein was examined in the upstream sequences of these genes using INO80 antibody in HEK293T cells. IgG antibody was used as the negative control for ChIP experiment. The negative (-ve) and positive (+ve) indicate the PCR controls. Input is 20% of sonicated chromatin. (C) Quantitative PCR for putative INO80 targets following ChIP assay in HEK293T cells. The Y axis shows the enrichment as percentage input observed. NAT2 and PSEN lack the INO80 binding motif. (*p value<0.05, **p value<0.001 Two tailed Student t-test)

We validated the association of INO80 with the regulatory regions of a subset of predicted target genes *in vivo*, by chromatin immunoprecipitation (ChIP) assay using anti-INO80 antibody. We used commercial antibodies (ab118787) and also peptide antibody from our lab [8]. We selected 14 genes, which belonged to [INO80 (-YY1)] subset of putative INO80 targets from the human genome. In concordance with our *in silico* predictions, we detected the association of INO80 with the upstream regions of these 14 genes in chromatin prepared from HEK293T mammalian cells (Fig 7). The homologues of these genes in *Drosophila* are experimentally validated targets of Polycomb and trithorax complexes. Twelve of these have putative INO80 binding motif in the promoter region and all of them showed association with INO80 in chromatin prepared from *Drosophila* embryos (Unpublished). We selected genes whose regulatory regions lack putative INO80 binding motif from NAT2 and PSEN1 as negative controls in immunoprecipitation reactions (Fig 7B). The ChIP followed by qPCR was carried out for some of these genes (Fig 7C). These results suggest the recruitment of the INO80 to the regulatory regions present upstream of its target genes. The sites of interaction in the whole genome are being determined by ChIP-seq approach.

### Distribution of INO80 around its consensus motif

**Fig 8:**
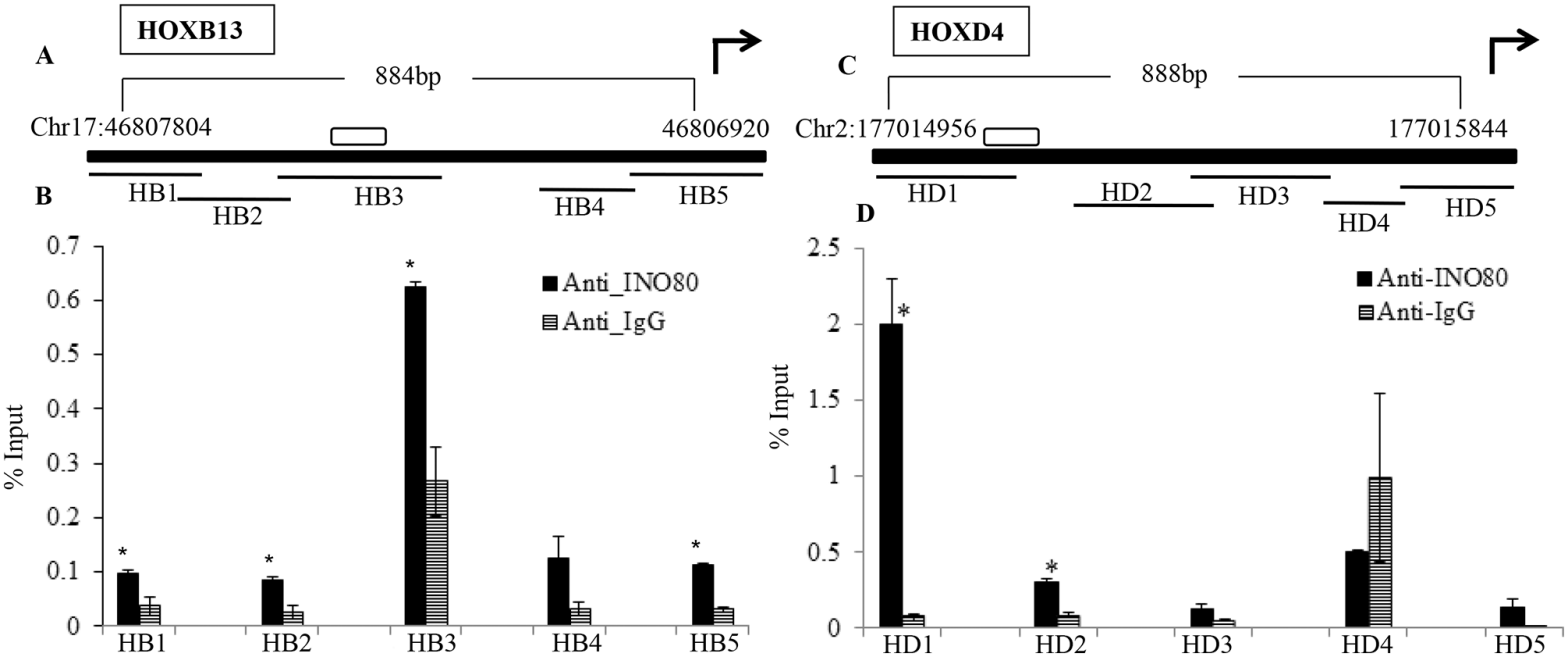
*In vivo* localization of INO80 in the upstream regions of its target genes. The data from ChIP followed by qPCR for HOXB13 (A & B) and HOXD4 (C & D) are shown. A & C- Line diagram indicating the upstream region analysed by qPCR of ChIP DNA as thick black line mapping on chromosome17 and 2 for HoxB13 and HoxD4 respectively along with the genomic positions, the bent arrow indicates the direction of transcription of the gene, unfilled box is the hINO80 binding motif present in the upstream region. HB1-HB5 and HD1-5 are the amplicons originating from the upstream region covering 884bp and 888bp of HoxB13 and HoxD4 respectively using primers listed (S2B Table). B&D show the enrichment as % Input for each region HB1-HB5 (B) and HD1-HD3(C), (*p value<0.05 and **p<0.001 in comparison with IgG for each primer set).

**Fig 9:**
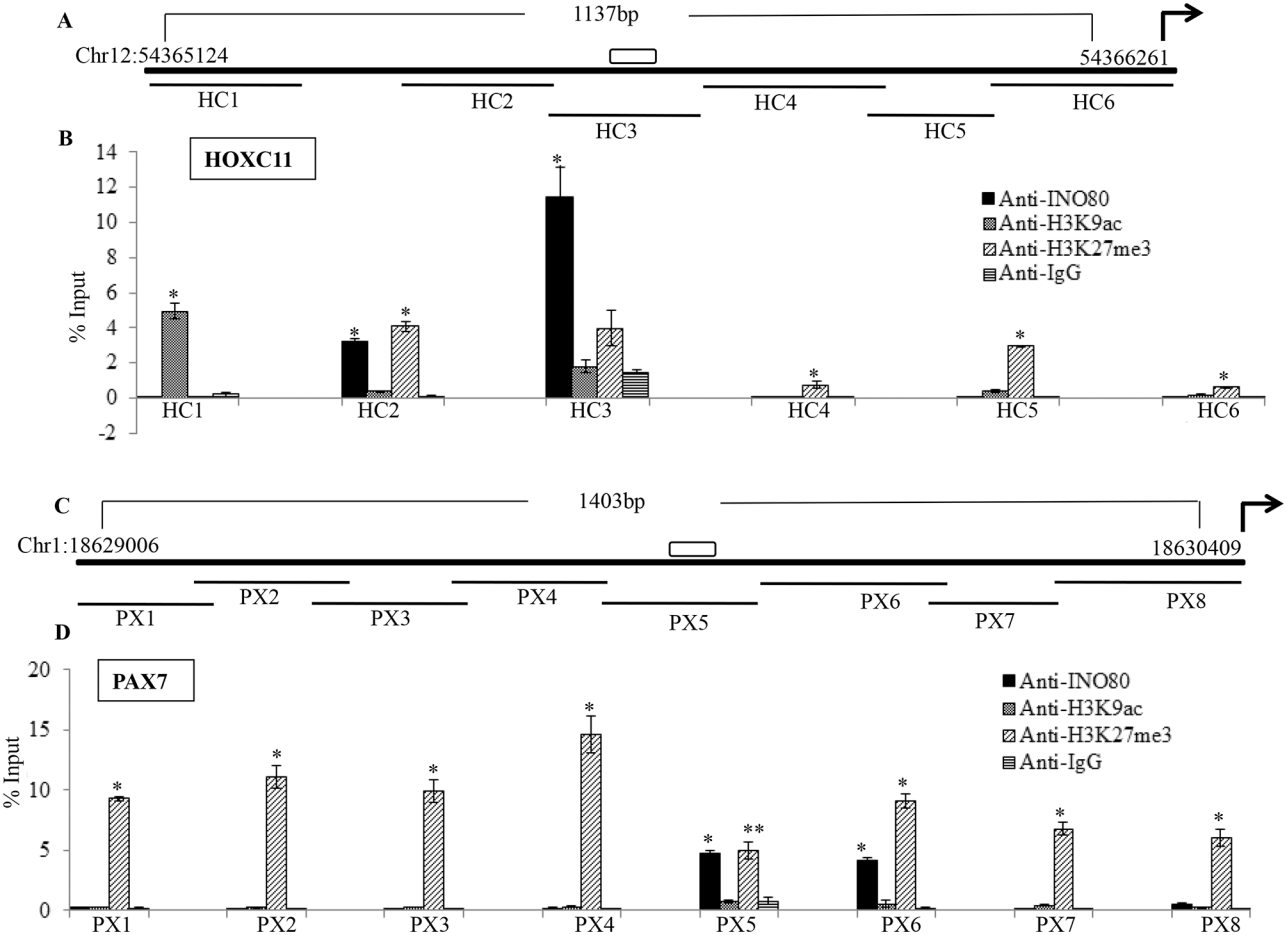
Localization of INO80 and histone marks in the upstream regions. The data from ChIP followed by qPCR for HOXC11(A & B) and PAX7(C & D) are shown. A & C- Line diagram indicates the upstream region analyzed by qPCR of ChIP DNA (thick black line) mapping on chromosome12 and 1 respectively for *HOXC11* and *PAX7*, along with the genomic positions; the bent arrow indicates the direction of transcription of the gene, and unfilled box is the hINO80 binding motif present in the upstream region. HC1-HC6 and PX1-8 are the amplicons originating from the upstream region covering 1137bp and 1403bp of HOXC11 and PAX7 respectively using primers listed (S2B Table). B&D show the enrichment as % Input for each region HC1-HB6(B) and PX1-PX8 (C) of INO80 and the H3K9me3 and K3K27me3 marks as shown in the inset (*p value<0.05 and **p<0.001 in comparison with IgG for each primer set).

The interaction of INO80 was examined in the vicinity of its consensus binding motif by qPCR using different primers following ChIP with anti-INO80 antibody (Figs 8 and 9). The total region of around 1kb including the consensus binding motif was analysed for INO80 enrichment. The length of each fragment assayed was approximately 200 base pairs, some of the amplicons in each gene have an overlap of 10–20 base pairs as indicated in the line diagram with the data. The sequence of the primers is listed in S2B Table. The occupancy of INO80 protein was checked in the regulatory region of HOXC11, HOXB13, HOXD4 and PAX7 genes. We observe the maximum enrichment of INO80 protein in region spanning the consensus binding motif: the amplicon HB3 in case of HOXB13, HD1 in case of HOXD4, HC3 in case of HOXC11 and PX5 in case of PAX7 (Figs 8 and 9). In some cases as in HD4 region of HOXD4 the binding of anti-INO80 antibody is not significant relative that with IgG. The region PX6 within PAX7 gene is very close to the binding motif and the enrichment observed in the region gene could arise due to the variable pattern of fragmentation of the cross-linked chromatin during sonication (Fig 9). In general it was observed that the regions flanking the binding motif are not enriched in INO80 protein. This indicates that the binding motif of INO80 protein could be mediating its interaction *in vivo* also.

### Analysis of epigenetic modifications around INO80 interaction site

The reporter assays indicate a negative effect of INO80 on transcription. We have examined the expression of the chosen target genes HEK cells and find that relative to GAPDH, the expression is low for these genes (S5 Fig). Further, we examined the activating and repressing histone marks around the INO80 binding region of HOXC11 and PAX7 genes by ChIP with anti-H3K9ac and anti-H3k27me3 followed by qPCR. In both the cases studied, INO80 enrichment was correlated with H3K27me3, the repressive mark, but not H3K9ac, the signature for active genes (Fig 9). The association of the INO80 interaction site with repressive histone mark was significant. The repressive epigenetic signature in this region correlates with interaction of INO80 protein.

## Discussion

The chromatin remodeling proteins belonging to the SNF2 family play a pivotal role in transcriptional regulation at promoters of a wide variety of genes. The specificity of targeting the ubiquitously expressed SNF2 members is achieved either through interaction of specific domains of the remodelers with modified histones or through the association of DNA-binding factors in the complex [10]. Therefore the detection of a highly conserved potentially DNA binding domain in INO80 prompted us to examine if INO80 shows any DNA sequence preference for binding [7]. We have earlier demonstrated the interaction of cloned DBINO domain with DNA *in vitro* by EMSA as well as UV-spectroscopy and here we detect the DNA sequence preference of INO80 [8]. We find that approximately 20% of the protein coding genes of the human genome contain INO80 binding motif with an over representation of metabolic and cellular process within this set.

The Ino80 complex in fission yeast interacts with Iec1, which contains zinc finger motifs similar to Ying Yang protein (YY1) and it recruits Ino80 on target genes on phosphate starvation [35]. The pleiohomeotic (PHO) protein in *Drosophila melanogaster* and Ying Yang protein (YY1) in human, the homologs of each other characterized as DNA binding proteins, were detected in the INO80 complex [11,24]. YY1 contains a domain called the REPO domain that is important for its DNA binding activity [36].

The highly conserved DBINO domain was identified in 2004 [7]. Based on the presence of this domain in all the INO80 homologs across phyla, phylogenetically INO80 clusters as a distinct sub-family, which was designated as the INO80 subfamily of SNF2 superfamily [5,7]. More recently, the presence of a domain named HSA (Helicase-SANT Associated) at the N-terminal end of hINO80 has been described [37]. HSA domain has nearly complete overlap with DBINO domain. The HSA domain of INO80 is shown to be involved in nucleosome interaction through Actin, Arp8 and Arp4 [38,39]. However independent binding of INO80 through HSA domain has not been reported. The presence of DNA binding domain in chromatin remodelers like CHD1 is identified at C-terminal end [40,41]. The recent crystal structure of the DNA binding domain of CHD1 has shown the structural similarity between the SANT-SLIDE domain of ISWI and the C-terminal sequence of CHD1 despite a lack of sequence similarity [42,43]. However CHD1 does not show sequence specific DNA binding [43]. Recent data on the crystal structure of INO80 complex of yeast, suggests the modular interaction of INO80 with different partners of the complex [38].

The functions of the INO80 complex include its role in transcriptional regulation, through its interaction with nucleosomes. Our results show that INO80 interacts with a 11mer motif distinct from that recognized by PHO/YY1 in the human genome. The *in vitro* interaction is sensitive to sequence variation in the conserved stretch (5’GTCAGCC’), indicating the specificity of the interaction. The involvement of INO80 in these interactions is demonstrated by the effect of knock-down of INO80 through siRNA in addition to supershift with anti-INO80 antibody.

Moshkin et al [44] carried out ChIP-chip analysis in S2 cells of *Drosophila melanogaster* with different chromatin remodelers including INO80. They analyzed the regions of 300-440bp. These regions were found to be AT rich and nucleosome dense. It is well known that DNA bending is required for nucleosome formation and thus AT rich regions favour nucleosome formation and thus nucleosome dense regions are generally AT rich. The DNA chip used by Moshkin et al [44] included the repetitive DNA and the heterochromatic regions (Affymetrics GeneChipArray 2.0R). In relation to this our motif length is 11 base pairs and the GC content in this short sequence would not reflect the base composition of the larger region of the genome. The transcription regulation through sequence specific binding by INO80 is only one of its additional functions depending on the partner proteins that are associated with INO80 in the complex.

Though the interaction of YY1/PHO with INO80 is detected, the implication of this interaction is not clearly known, except in the case of mouse Ino80, where it is shown that knock-down of either Yy1 or Ino80 affects homologous recombination mediated repair [24,45,46]. Therefore, it appears that the interaction with a recruiter is not essential for the chromatin remodeling functions of INO80 or its role in H2A.Z replacement [47]. We examined the functional implication of the interaction of INO80 with DNA by studying its effect on reporter expression. The observed down-regulation of the reporter expression caused by the consensus DNA binding motif is dependent on INO80, but not on YY1 which is known to be its recruiter [45]. However when the INO80 binding motif is cloned downstream to the reporter gene, there is significant repression observed but this effect is not dependent on the presence of INO80, YY1 or the other members of the PRC2 complex that we tested. This shows the specificity of the INO80 dependent regulation of the reporter gene we observe from the upstream region. In this context it is relevant to point out that INO80 exerts regulatory effect on homeotic genes in *Drosophila melanogaster* and is essential for completion of development [26]. We detect the mis-expression of homeotic genes in INO80 knock-out lines and also in INO80 RNAi lines [28]. In addition we have the evidence of enrichment of dIno80 at the bxd and iab-7 PREs in *Drosophila melanogaster* (Unpublished). In the present case, in addition to YY1, we did not observe any effect of other PRC members like SUZ12 and EED on reporter expression, thus suggesting that either INO80 by itself or a different complex of INO80 could be involved in the regulatory function. In addition, the identification of INO80 motif in the upstream region of genes without the co-occurrence of YY1 binding site, strongly suggests that INO80 might have YY1 independent function in transcriptional regulation. In *D*. *melanogaster*, we have demonstrated that dIno80 plays a role in early development independent of Pho, the *Drosophila* homologue of YY1 [28].

**Fig 10:**
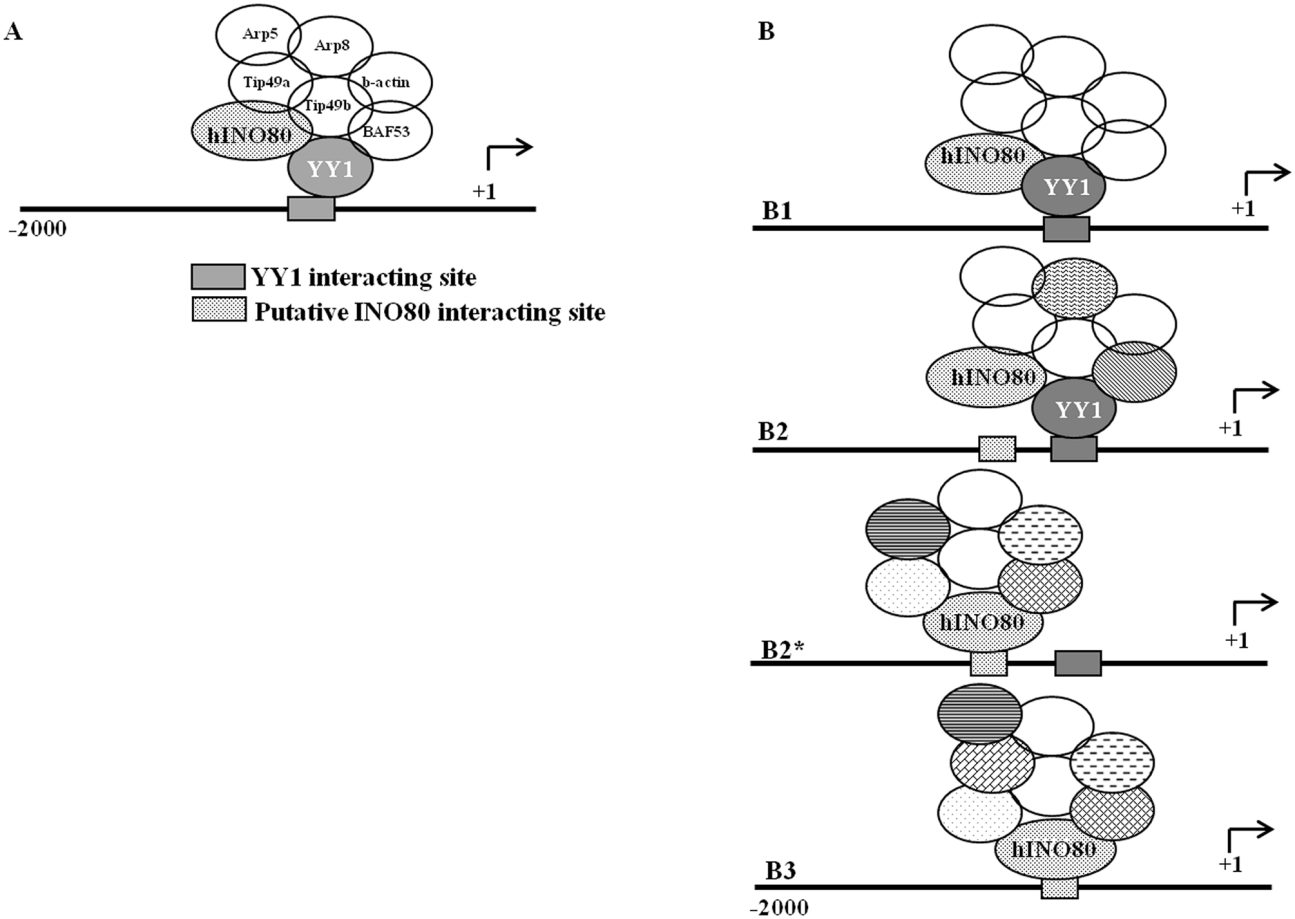
A model for interaction of INO80 with its target sites. The binding motif for YY1 and INO80 are indicated as rectangular boxes; +1 and the arrow indicate the transcription start site and the direction of transcription, A-the known hINO80-YY1 complex involved in chromatin remodeling [11,36]. B- represents four possible scenario for the interaction of INO80 in the upstream region of the genes that it regulates; B1-is a complex recruited by YY1 in which INO80 could be a partner; B2 & B2*- represent genomic regions where both YY1 and INO80 binding motif is present in close vicinity and the recruitment may be through either of them and two different complexes interacting at the same genomic region is possible; B3- represents a region where only INO80 binding motif is present.

INO80 is associated with the regulatory regions of its putative target genes that are devoid of YY1 binding sites in their regulatory regions, thus indicating that INO80 could act as DNA binding partner/recruiter of transcription regulatory complexes. The possible contextual interactions of INO80 are diagrammatically represented in Fig 10. However, it remains to be investigated if there are complexes containing INO80 but devoid of YY1 and if the recruitment function is shared by both the proteins depending on the cis-elements present at the given locus.

Based on our results, we propose that the DNA-binding activity of INO80 itself can mediate the recruitment of a regulatory complex in certain contexts. Our *in silico* analysis has shown the independent occurrence of INO80 binding motif as well as its co-occurrence with YY1 motif in the human genome. We speculate that a novel complex could be involved in these interactions as there is no effect of knock-down of the members of PRC2 complex on the repression of the reporter gene.

It is interesting to note that the region upstream of the genes we have analyzed is enriched in repressive mark H3K27me3 compared to the activating mark, H3K4me3 in the ENCODE data (https://genome.ucsc.edu/ENCODE). Our results are consistent with the previous reports that establish the involvement of Ino80 in transcriptional silencing in yeast [1]. Ino80 plays a critical role in regulating the ecdysone-induced genes in *Drosophila* [27]. It causes transcriptional repression of these genes during prepupal development. It was observed, that mutant Ino80 caused longer prepupal stage, whereas overexpression of Ino80 leads to shortening of the prepupal stage. It is also shown in *Drosophila* S2 cells that, INO80 complex increases the nucleosome density at the target loci [44]. The Ino80 complex also serves as a silencing complex in *Saccharomyces cerevisiae* restricting the transcription to genic regions in euchromatin. It demarcates the units of transcription across the genome, confining the activity to gene bodies and away from the silent regions (heterochromatin). It cooperates with the Sir complex to maintain gene silencing at heterochromatin region [48].

The DBINO domain we identified by in silico analysis has been detected in several proteins and not all of them belong to the SNF2 ATPase/helicase family members. In the data given at Interpro site (www.ebi.ac.uk/interpro/), DBINO domain is detected in more than 740 proteins, among these there are DNA dependent ATPase family member, INO80 and also proteins with other functions, including transcription factors. This suggests that DBINO domain could be a novel functional domain in proteins. The HSA domain region has structural similarity with the DNA interacting domain of ISWI/CHD1, even if there is no amino acid sequence conservation, as in the case of CHD1 and ISWI [39]. This domain so far is not known to have any sequence specific DNA binding activity. In *Saccharomyces cerevisiae*, it is reported that the deletion of a highly conserved region towards the N-terminal of the ATPase domain abolished DNA binding as well as Arp interaction with Ino80 [49]. The deleted region overlaps with DBINO domain that we have identified.

In conclusion, the role of DNA-binding domain of INO80 on one hand and its well known DNA dependent ATPase activity on the other is probably indicative of the dual function to INO80 protein at a subset of INO80 target sites. Though there is no direct evidence, the presence of other DNA dependent ATPases in INO80 complex like RUVBL1 and RUVBL2 in both human and *Drosophila* makes it possible that in some cases INO80 functions as a recruiter through its DBINO domain apart from or in addition to being a DNA dependent ATPase [11, 24].

## Supporting Information

S1 Fig Detection of INO80. The anti-INO80 antibody was generated against the DBINO domain in rabbit. The IgG fraction was tested with DBINO domain with protein extracts prepared from E.coli transformed with pGEX-DBINO before (UI) and after IPTG induction (I) following electrophoresis in 10%SDS PAGE, A-probed with Anti-GST and B-Anti-INO80 antibody, C-INO80 from nuclear extracts from HEK293T cells were detected with Anti-INO80 antibody (Abcam). 5 & 10μg of the protein extracts were resolved on 8% SDS-PAGE and probed with 1:100 dilution of anti-INO80 antibody. M: Prestained protein ladder (MBI Fermentas).
(TIF)

S2 Fig Strategy used for identification of INO80 binding motif. (A) Diagrammatic representation of the workflow. (B) The generalized sequence of the oligonucleotides constituting the random oligonucleotide library, (N)_20_ is the random sequence.
(TIF)

S3 Fig Western blot showing the knock down of INO80 protein. The knock down was carried out by transfecting siRNA against INO80 and checking the protein level after 24 and 48 hrs respectively. The level of knock down was around 50% after 48 hrs. The PCNA protein was used as a loading control.
(TIF)

S4 Fig Workflow followed for predicting INO80 and YY1 target genes on the human genome and their distribution. [INO80(-YY1)] represents the subset of putative INO80 targets devoid of YY1 binding sites; [INO80(+YY1)] represents the subset of the list having both INO80 and YY1 binding sites in the upstream sequences (2000bp upstream of transcription start site).
(TIF)

S5 Fig Expression of target genes in HEK cells. Quantitative PCR carried out for expression status of target genes tested for INO80 interaction. The reciprocal of Ct values are plotted. Relative to GAPDH the other genes have low expression.
(TIF)

S1 Table Sequence of oligonucleotides (33mers) used in EMSA experiments. (DOC)

S2 Table (A)List of primers used in ChIP assays with Anti-INO80 antibody for putative targets in the human genome. (B) List of primers used in the ChIP experiment with antibody against INO80 and modified histone and the corresponding amplicon size in partial tiling assay. (DOC)

S3 Table Sequences of random oligonucleotides obtained from individual clones following SELEX. (DOC)

S4 Table The occurrence of INO80 and YY1 protein binding sequence motif in the human genome. The numbers of putative sites of interaction of INO80 and YY1 proteins in the regions upstream of protein coding genes were analyzed. The sequences of YY1 motif we used for analysis are: 5’GCCATCAT3’ (8mer) and 5’CCGCCATNTT3’ (10mer) (www.genecards.org).
(DOCX)

S5 Table List of putative INO80 targets in experimentally validated for PRC1-PRC2 binding in mammals. (DOC)
