# Supplementary figures and images for: Interaction of the Chromatin Remodeling Protein hINO80 with DNA
Source: PLoS One. 2016 Jul 18;11(7):e0159370. doi: 10.1371/journal.pone.0159370 (PMC4948845; doi:10.1371/journal.pone.0159370)

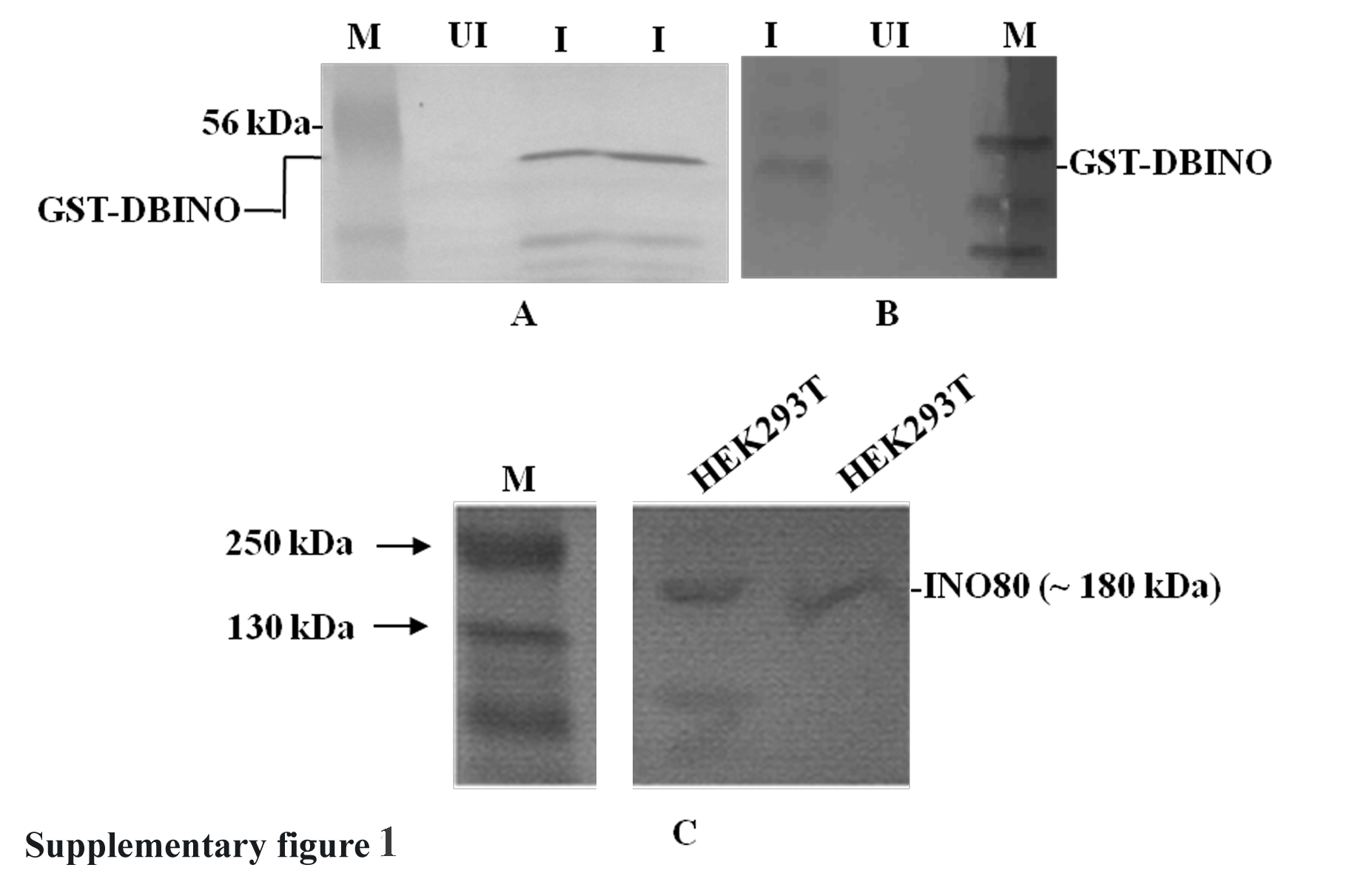

Supplement: S1 Fig — The anti-INO80 antibody was generated against the DBINO domain in rabbit. The IgG fraction was tested with DBINO domain with protein extracts prepared from E.coli transformed with pGEX-DBINO before (UI) and after IPTG induction (I) following electrophoresis in 10%SDS PAGE, A-probed with Anti-GST and B-Anti-INO80 antibody, C-INO80 from nuclear extracts from HEK293T cells were detected with Anti-INO80 antibody (Abcam). 5 & 10μg of the protein extracts were resolved on 8% SDS-PAGE and probed with 1:100 dilution of anti-INO80 antibody. M: Prestained protein ladder (MBI Fermentas). (TIF) [file pone.0159370.s001.tif]

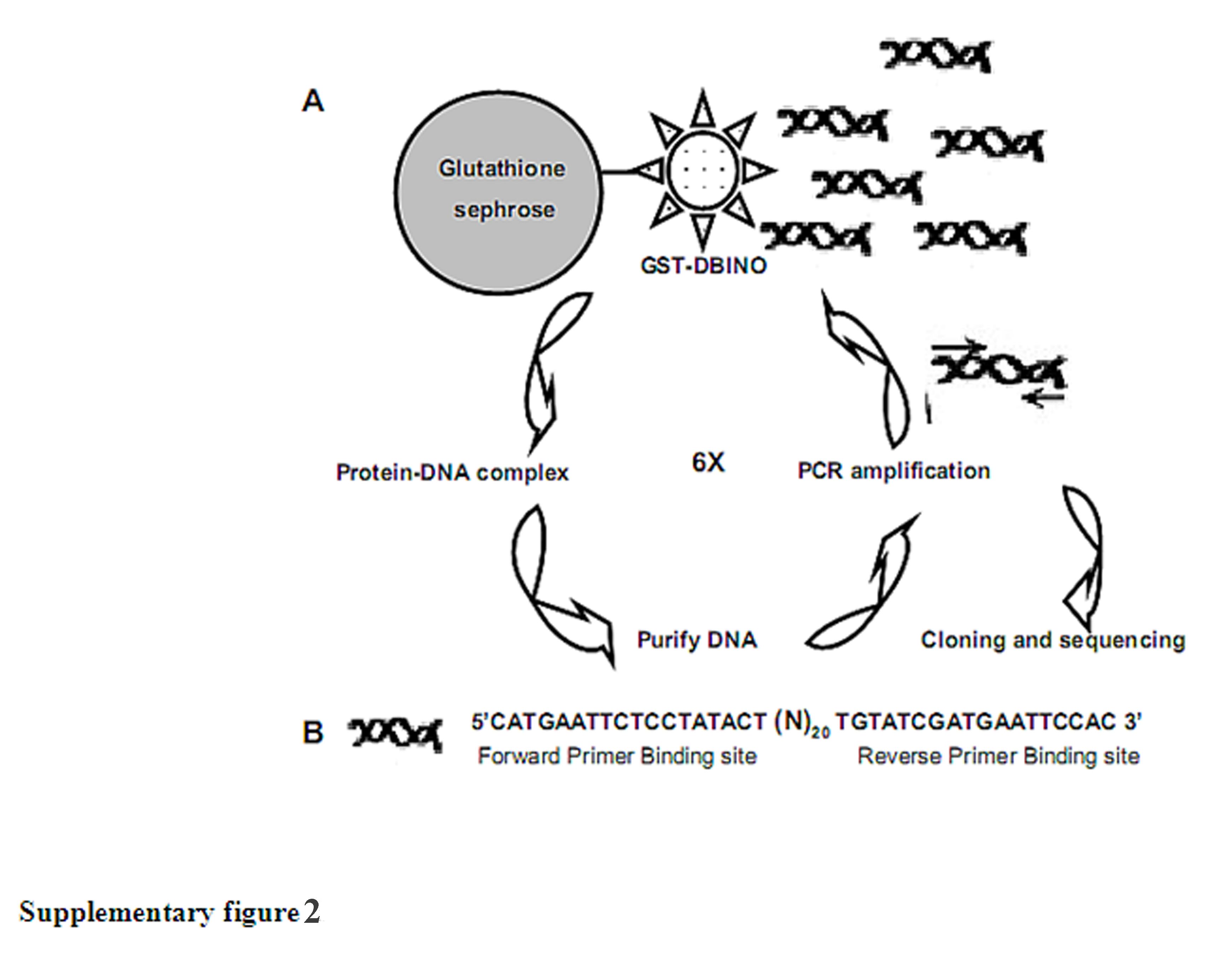

Supplement: S2 Fig — (A) Diagrammatic representation of the workflow. (B) The generalized sequence of the oligonucleotides constituting the random oligonucleotide library, (N)20 is the random sequence. (TIF) [file pone.0159370.s002.tif]

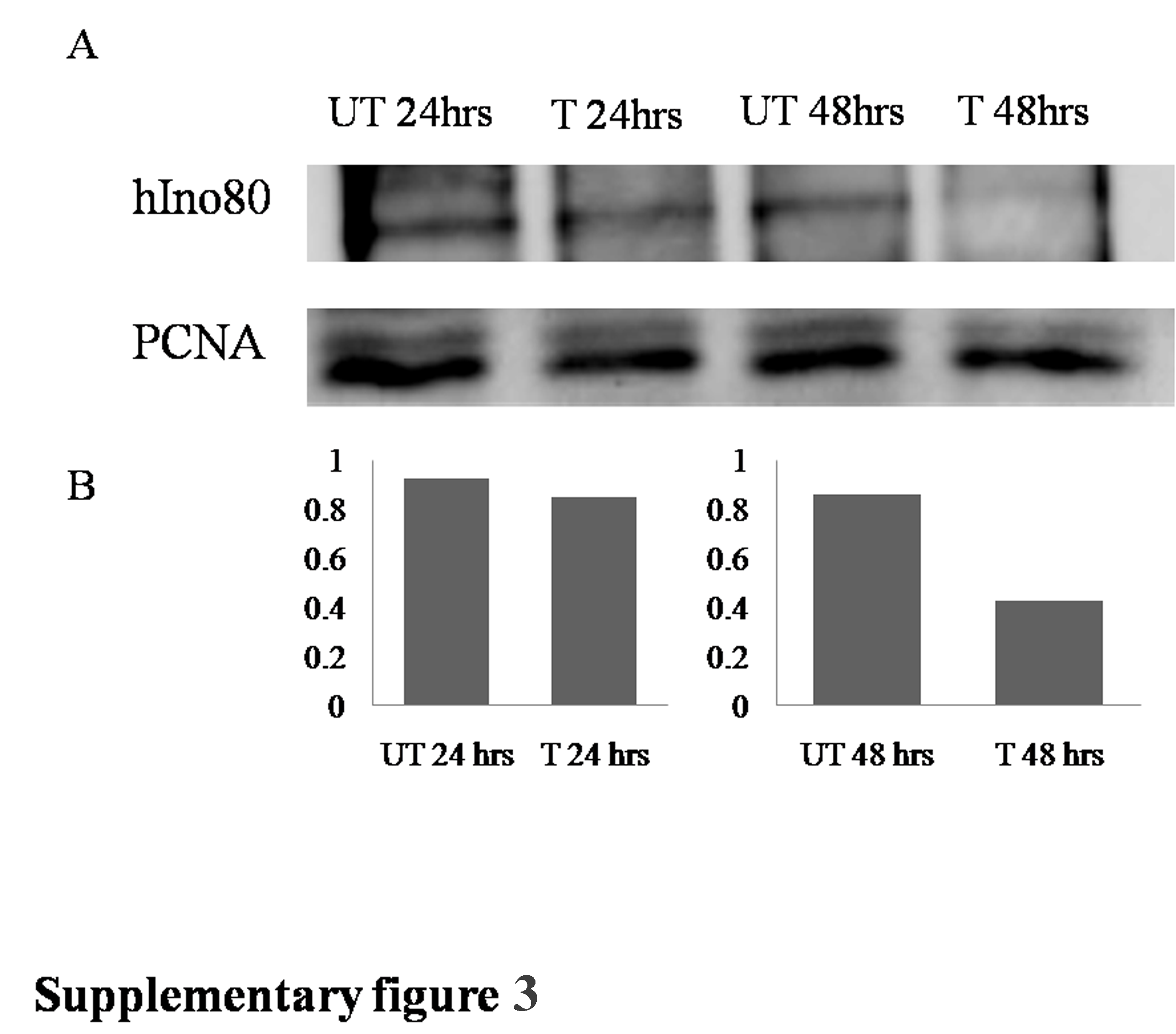

Supplement: S3 Fig — The knock down was carried out by transfecting siRNA against INO80 and checking the protein level after 24 and 48 hrs respectively. The level of knock down was around 50% after 48 hrs. The PCNA protein was used as a loading control. (TIF) [file pone.0159370.s003.tif]

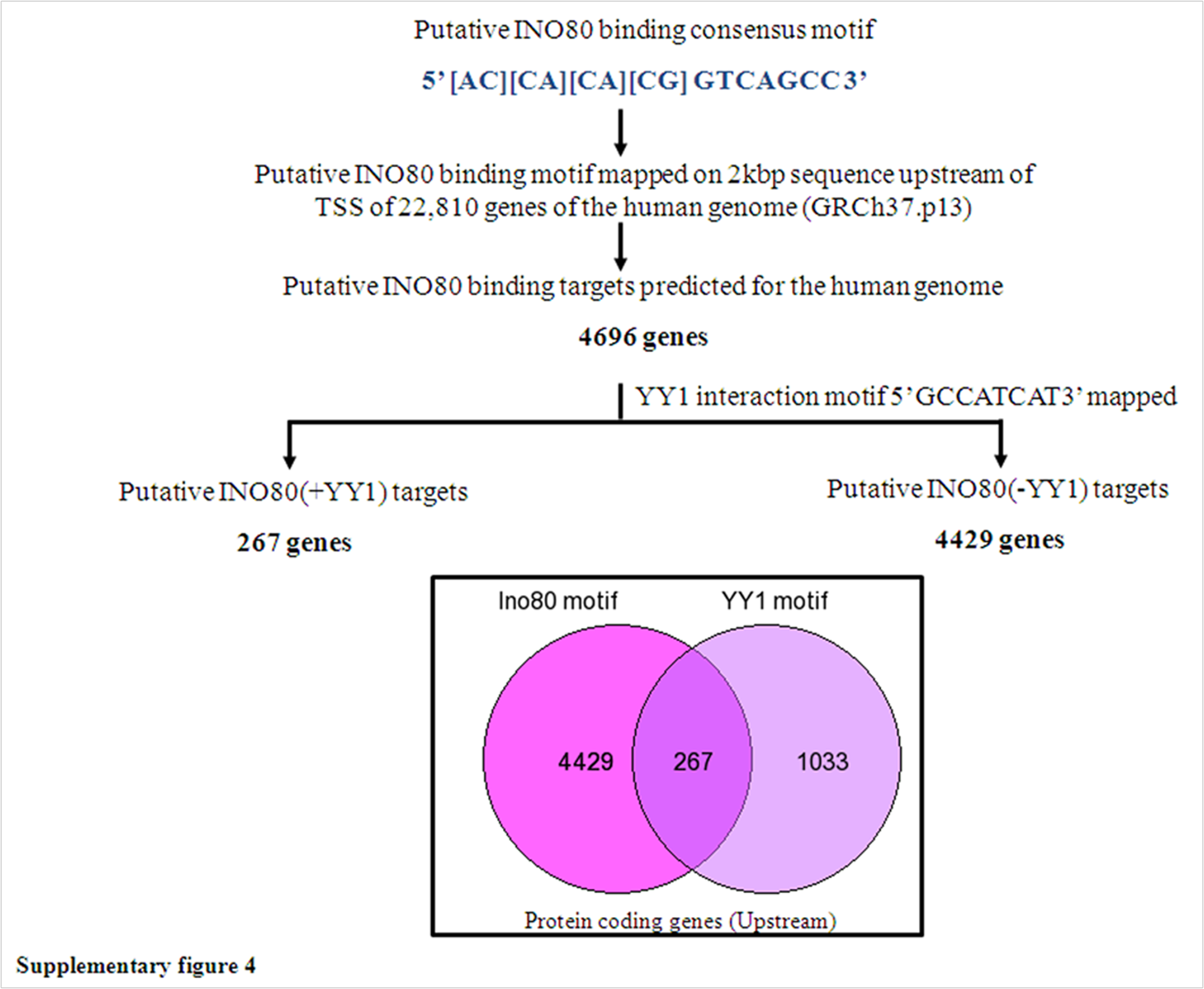

Supplement: S4 Fig — [INO80(-YY1)] represents the subset of putative INO80 targets devoid of YY1 binding sites; [INO80(+YY1)] represents the subset of the list having both INO80 and YY1 binding sites in the upstream sequences (2000bp upstream of transcription start site). (TIF) [file pone.0159370.s004.tif]

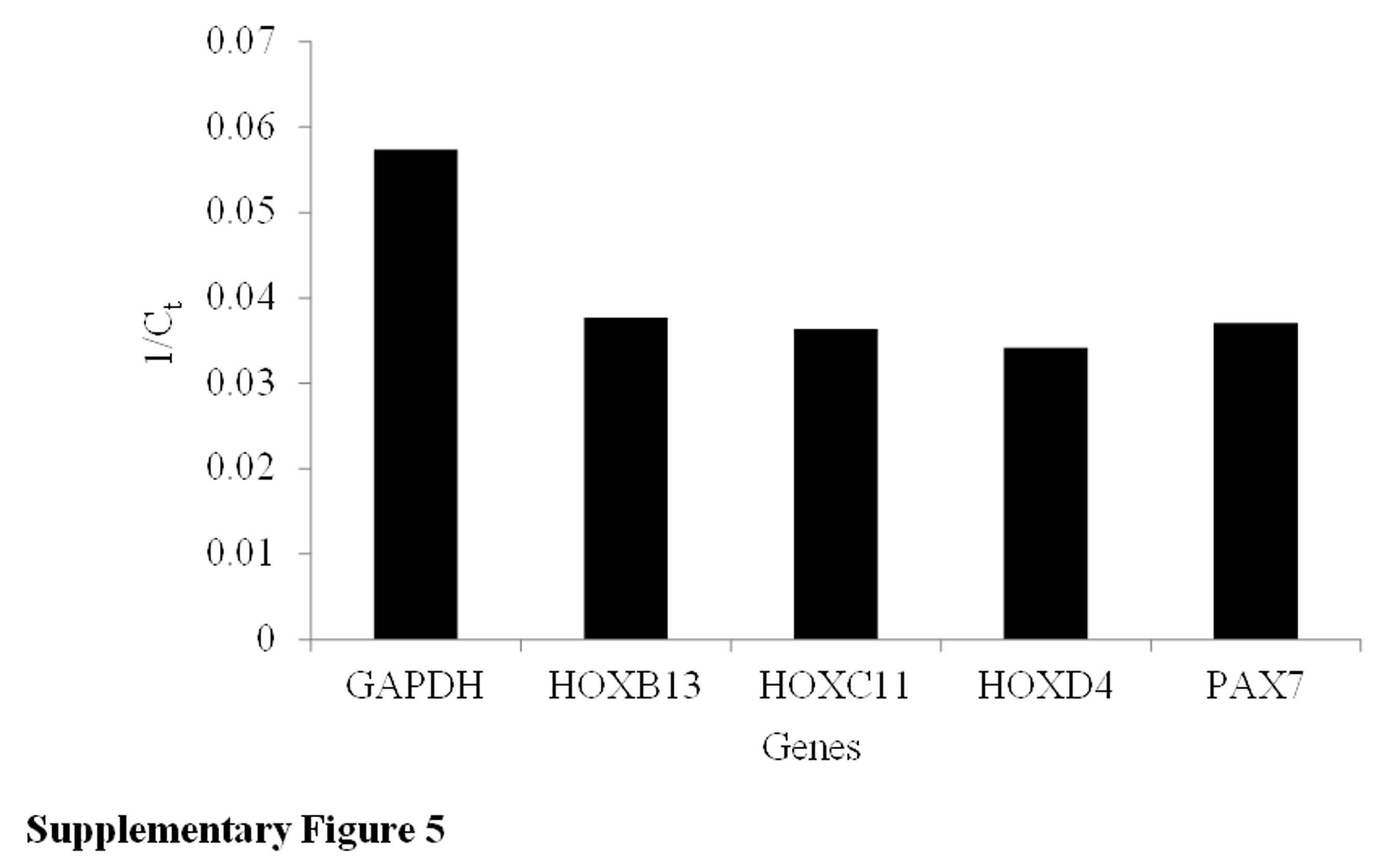

Supplement: S5 Fig — Quantitative PCR carried out for expression status of target genes tested for INO80 interaction. The reciprocal of Ct values are plotted. Relative to GAPDH the other genes have low expression. (TIF) [file pone.0159370.s005.tif]
