# Supplementary material for: Interaction of the Chromatin Remodeling Protein hINO80 with DNA
Source: PLoS One. 2016 Jul 18;11(7):e0159370. doi: 10.1371/journal.pone.0159370 (PMC4948845; doi:10.1371/journal.pone.0159370)
Supplement: S1 Table — (DOC) [file pone.0159370.s006.doc]

| Oligos | Sequence |
| --- | --- |
| Ino80 binding motif (3 repeats) | CCCCGTCAGCCCCCCGTCAGCCCCCCGTCAGCC |
| Ino80 binding motif (3 repeats)  (Reverse complement) | GGCTGACGGGGGGCTGACGGGGGGCTGACGGGG |
| Non-specific | CCGCCATCTTCTCCTGAGCATCTTCTCCTGAGC |
| Non-specific (Reverse complement) | GCTCAGGAGAAGATGCTCAGGAGAAGATGGCGG |
| M1 | CCCCGTCAG**TT** CCCCGTCAG**TT** CCCCGTCAG**TT** |
| M2 | CCCCGTCA**T**CC CCCCGTCA**T**CC CCCCGTCA**T**CC |
| M3 | CCCCG**G**CAGCC CCCCG**G**CAGCC CCCCG**G**CAGCC |
| M4 | CCCC**T**TCAGCC CCCC**T**TCAGCC CCCC**T**TCAGCC |
| M5 | CCCCGTC**C**GCC CCCCGTC**C**GCC CCCCGTC**C**GCC |
| M6 | CCCCG**A**CAGCC CCCCG**A**CAGCC CCCCG**A**CAGCC |

Supplementary Table1. Sequence of oligonucleotides (33mers) used in EMSA experiments.
