# Supplementary material for: Interaction of the Chromatin Remodeling Protein hINO80 with DNA
Source: PLoS One. 2016 Jul 18;11(7):e0159370. doi: 10.1371/journal.pone.0159370 (PMC4948845; doi:10.1371/journal.pone.0159370)
Supplement: S2 Table — (DOC) [file pone.0159370.s007.doc]

**Supplementary Table 2: (A) List of primers used in ChIP assays with Anti-INO80 antibody for putative targets in the human genome .**

**(B)** List of primers used in the ChIP experiment with antibody against INO80 and modified histone and the corresponding amplicon size in partial tiling assay.

A)

| **Gene** | **Primer name** | **Primer sequence (5' - 3')** | **Annealing temperature(°C)** |
| --- | --- | --- | --- |
| HOXB13 | HOXB13 FP | ACACAGGCGTCTGTATTC | 54 |
|  | HOXB13 RP | GACAGCAACACAAAGAGG |  |
| HOXC11 | HOXC11 FP | ATGTGTCCTGTGGTGCTC | 52 |
|  | HOXC11 RP | GCCACTCCCTAGTCAAAG |  |
| HOXA1 | HOXA1 FP | GTTCTATGCGCTCCTCAC | 52 |
|  | HOXA1 RP | TCCGCAGTGATGGATCAC |  |
| HOXD4 | HOXD4 FP | TTTGTGTGGTATCCGTATAG | 54 |
|  | HOXD4 RP | GCTGCTCTATGTCACCAG |  |
| CACNA1 | CACNA1 FP | GACCTGGGTTTCGTCTTTC | 50 |
|  | CACNA1 RP | AATAACCGAGGAAGGAATC |  |
| PAX7 | PAX7 FP | GCAAAGGTCACAGGTGAG | 52 |
|  | PAX7 RP | ACCAGTAGCCTCAACCTC |  |
| LHX3 | LHX3 FP | CCTGCTGCTTCGTGTCTCAC | 52 |
|  | LHX3 RP | TGAAGCTGGAGACGGTAAAC |  |
| BARHL1 | BARHL1 FP | AAATCGCCGGTAAACAGTTG | 54 |
|  | BARHL1 RP | GGCTCATGACTCTCCACCTG |  |
| FOXA1 | FOXA1 FP | TAGTTTCTGACACACAATTCG | 55 |
|  | FOXA1 RP | CCAAACAAATCTCTTAAGTC |  |
| OSR1 | OSR1 FP | CTCCAAGAGTCACTCAGCAG | 53 |
|  | OSR1 RP | TCTGAGTCAAGGAGCTGGTTC |  |
| SIX3 | SIX3 FP | CACATAGGCAGAAAGAGGC | 54 |
|  | SIX3 RP | CTTTGCAACTCTTAATCTCG |  |
| NKX2-8 | NKX2-8 FP | AATTCCTGGGTGCTAATAAAC | 50 |
|  | NKX2-8 RP | GTCTGATTAGCCCAAAGTG |  |
| HOXD8 | HOXD8 FP | AACGTTTCTGTCCGCTCTTC | 52 |
|  | HOXD8 RP | AACGTTTCTGTCCGCTCTTC |  |
| OTX1 | OTX1 FP | CGCGGTGTCTTTGTTCTG | 54 |
|  | OTX1 RP | CCGGGAGATCTTCTTAGG |  |
| NAT2 | NAT2 FP | ACCAAATGTCAATCAGGATATG | 52 |
|  | NAT2 RP | CAAATCTTTGTCTTGTGGC |  |
| PSEN1 | PSEN1 FP | TTGAGACATAGTTTCGCTC | 54 |
|  | PSEN1 RP | AAATATGGTACAATAGGCAG |  |
| PAX7 | PX1 FP | CTGCACCTTGACACACAGACC | 60 |
|  | PX1 RP | GCTGTCCTCTGCCTGGAAG |  |
| PAX7 | PX2 FP | CAGGGAAGAAGTGCCTTGA | 60 |
|  | PX2 RP | GCTTTGACCTCCATAGGTGG |  |
| PAX7 | PX3 FP | CTATGGAGGTCAAAGCAGGC | 60 |
|  | PX3 RP | ATTGGAAGGGGGACACCG |  |
| PAX7 | PX4 FP | CGAAAGCTACTTGGCATC | 60 |
|  | PX4 RP | CTTCGCCTCACCTGTGAC |  |
| PAX7 | PX5 FP | CATGCTGCAAGGGGCAAAG | 60 |
|  | PX5 RP | CAGGCCTTTTGTCTCCCGG |  |
| PAX7 | PX6 FP | GTGAGATTGAGGGCTGGG | 60 |
|  | PX6 RP | AGTCAGGGTGGGGTGATC |  |
| PAX7 | PX7 FP | CATTCAGTTGGGGCGTCC | 60 |
|  | PX7 RP | CCAGGCCGGGCCTATAAC |  |
| PAX7 | PX8 FP | GCTGGAGACGTCTGCACG | 60 |
|  | PX8 RP | CTCTACCTTCCTGCCGCTC |  |
| HOXC11 | HC1 FP | CACCCTGAGAATGGGACCC | 60 |
|  | HC1 RP | CATGATAAGGTGCTGCGGC |  |
| HOXC11 | HC2 FP | CTTCGGTTCTTCCCCTCC | 60 |
|  | HC2 RP | CCCAACCCCTTGCCTTCCA |  |
| HOXC11 | HC3 FP | GCCCACATGTGTCCTGTG | 60 |
|  | HC3 RP | CCCTAGTCAAAGCCCCCAG |  |
| HOXC11 | HC4 FP | TAAGATCTGGTCACAGCC | 60 |
|  | HC4 RP | GGGACTAGGTTTGGGGTTC |  |
| HOXC11 | HC5FP | CCCCAAACCTAGTCCCC | 60 |
|  | HC5 RP | GTGAAGAAAGCTACATCCACTG |  |
| HOXC11 | HX6 FP | GGATTTGAAAATCCACAGTGG | 60 |
|  | HC6 RP | CCCTGGCTAGGGAGTCAG |  |
| HOXB13 | HB1 FP | GACAAGCAAGAAACTCTGGTG | 60 |
|  | HB1 RP | GCCCACAGGCAGTAGTGTAC |  |
| HOXB13 | HB2 FP | GTGGCTGGGGTTTCCCCTTC | 60 |
|  | HB2 RP | GCCAGTCTCCCTGCCCTG |  |
| HOXB13 | HB3 FP | ACCATAGGGAGAGGACTG | 60 |
|  | HB3 RP | TCCTGAAGTGTCAGTCAG |  |
| HOXB13 | HB4 FP | CCGTCCCTCCGTATGTCTTTAC | 60 |
|  | HB4 RP | CCCTGATTCGGGTGAGGCT |  |
| HOXB13 | HB5 FP | CTACGTTCGTGTCTCCCG | 60 |
|  | HB5 RP | GGGTCCTGGGTACTAGCA |  |
| HOXD4 | HD1 FP | CTGCGTCGAACGGTGGTG | 60 |
|  | HD1 RP | GAAGTTTTTGCATCGACC |  |
| HOXD4 | HD2 FP | CTGGTGACATAGAGCAGC | 60 |
|  | HD2 RP | TGGCCTTGTACCTCACTC |  |
| HOXD4 | HD3 FP | GGAGTGAGGTACAAGGCCAG | 60 |
|  | HD3 RP | GCACCCTAGAGCTCCCCA |  |
| HOXD4 | HD4 FP | GGCCTGTGGGAAGAAAGC | 60 |
|  | HD4 RP | GCTTTCTACCAGTGCCCAG |  |
| HOXD4 | HD5 FP | CAGCTTTGGCTGGGAACC | 60 |
|  | HD5 RP | GGGAGCAGGGATTCTGCC |  |

: B)

| **Gene** | **Primer name** | **Primer sequence (5' - 3')** | **Annealing temperature**  **(°C)** | **Amplicon size (bp)** |
| --- | --- | --- | --- | --- |
| PAX7 | PX1 FP | CTGCACCTTGACACACAGACC | 60 | 113 |
|  | PX1 RP | GCTGTCCTCTGCCTGGAAG |  |  |
| PAX7 | PX2 FP | CAGGGAAGAAGTGCCTTGA | 60 | 183 |
|  | PX2 RP | GCTTTGACCTCCATAGGTGG |  |  |
| PAX7 | PX3 FP | CTATGGAGGTCAAAGCAGGC | 60 | 168 |
|  | PX3 RP | ATTGGAAGGGGGACACCG |  |  |
| PAX7 | PX4 FP | CGAAAGCTACTTGGCATC | 60 | 183 |
|  | PX4 RP | CTTCGCCTCACCTGTGAC |  |  |
| PAX7 | PX5 FP | CATGCTGCAAGGGGCAAAG | 60 | 217 |
|  | PX5 RP | CAGGCCTTTTGTCTCCCGG |  |  |
| PAX7 | PX6 FP | GTGAGATTGAGGGCTGGG | 60 | 219 |
|  | PX6 RP | AGTCAGGGTGGGGTGATC |  |  |
| PAX7 | PX7 FP | CATTCAGTTGGGGCGTCC | 60 | 190 |
|  | PX7 RP | CCAGGCCGGGCCTATAAC |  |  |
| PAX7 | PX8 FP | GCTGGAGACGTCTGCACG | 60 | 135 |
|  | PX8 RP | CTCTACCTTCCTGCCGCTC |  |  |
| HOXC11 | HC1 FP | CACCCTGAGAATGGGACCC | 60 | 180 |
|  | HC1 RP | CATGATAAGGTGCTGCGGC |  |  |
| HOXC11 | HC2 FP | CTTCGGTTCTTCCCCTCC | 60 | 189 |
|  | HC2 RP | CCCAACCCCTTGCCTTCCA |  |  |
| HOXC11 | HC3 FP | GCCCACATGTGTCCTGTG | 60 | 200 |
|  | HC3 RP | CCCTAGTCAAAGCCCCCAG |  |  |
| HOXC11 | HC4 FP | TAAGATCTGGTCACAGCC | 60 | 198 |
|  | HC4 RP | GGGACTAGGTTTGGGGTTC |  |  |
| HOXC11 | HC5FP | CCCCAAACCTAGTCCCC | 60 | 186 |
|  | HC5 RP | GTGAAGAAAGCTACATCCACTG |  |  |
| HOXC11 | HX6 FP | GGATTTGAAAATCCACAGTGG | 60 | 208 |
|  | HC6 RP | CCCTGGCTAGGGAGTCAG |  |  |
| HOXB13 | HB1 FP | GACAAGCAAGAAACTCTGGTG | 60 | 162 |
|  | HB1 RP | GCCCACAGGCAGTAGTGTAC |  |  |
| HOXB13 | HB2 FP | GTGGCTGGGGTTTCCCCTTC | 60 | 182 |
|  | HB2 RP | GCCAGTCTCCCTGCCCTG |  |  |
| HOXB13 | HB3 FP | ACCATAGGGAGAGGACTG | 60 | 188 |
|  | HB3 RP | TCCTGAAGTGTCAGTCAG |  |  |
| HOXB13 | HB4 FP | CCGTCCCTCCGTATGTCTTTAC | 60 | 179 |
|  | HB4 RP | CCCTGATTCGGGTGAGGCT |  |  |
| HOXB13 | HB5 FP | CTACGTTCGTGTCTCCCG | 60 | 190 |
|  | HB5 RP | GGGTCCTGGGTACTAGCA |  |  |
| HOXD4 | HD1 FP | CTGCGTCGAACGGTGGTG | 60 | 184 |
|  | HD1 RP | GAAGTTTTTGCATCGACC |  |  |
| HOXD4 | HD2 FP | CTGGTGACATAGAGCAGC | 60 | 200 |
|  | HD2 RP | TGGCCTTGTACCTCACTC |  |  |
| HOXD4 | HD3 FP | GGAGTGAGGTACAAGGCCAG | 60 | 150 |
|  | HD3 RP | GCACCCTAGAGCTCCCCA |  |  |
| HOXD4 | HD4 FP | GGCCTGTGGGAAGAAAGC | 60 | 195 |
|  | HD4 RP | GCTTTCTACCAGTGCCCAG |  |  |
| HOXD4 | HD5 FP | CAGCTTTGGCTGGGAACC | 60 | 162 |
|  | HD5 RP | GGGAGCAGGGATTCTGCC |  |  |
