# Supplementary material for: Interaction of the Chromatin Remodeling Protein hINO80 with DNA
Source: PLoS One. 2016 Jul 18;11(7):e0159370. doi: 10.1371/journal.pone.0159370 (PMC4948845; doi:10.1371/journal.pone.0159370)
Supplement: S3 Table — (DOC) [file pone.0159370.s008.doc]

Supplementary Table 3: Sequences of random oligonucleotides obtained from individual clones following SELEX.

| **S.No.** | **Clone No.** | **Sequence (5'-3')** |
| --- | --- | --- |
| 1 | 6b,8b | CACAGAAACCGTCAGCCGGC |
| 2 | 2b | CACGGAAACCGTCAGCCGGC |
| 3 | 22 | CAACCGTCAGCCGTCCCATG |
| 4 | 1b | AAACACCCGTCAGCCTTTG |
| 5 | 36 | ACCCGTCAGCCGTCTCTTGT |
| 6 | 60 | CGCTCTCACCCGTCAGCCGG |
| 7 | 25b | GCAAAGCACAGGTCAGCCC |
| 8 | 12b | AATCACAGGTCAGCCCATTG |
| 9 | 10 | CCAGATTGGATCAGTCAGCC |
| 10 | 27 | CCTCGGTAGATCAGTCAGCC |
| 11 | 12d | TCTACAAAGTCAGCCTTTGC |
| 12 | 18a | CCACCTCCGTCAGCCTCCCG |
| 13 | 13a,16a | CCCGGCAAGGTCAGCCCTTG |
| 14 | 12a | CTCACGTCAGCCGCGTCTAT |
| 15 | 24 | TTACCTTACGTCAGCCGTC |
| 16 | 29 | CACTGTTCGGTCAGCCCTCC |
| 17 | 2a,6a,8a | ATGGTACCTCGTCAGCCTCG |
| 18 | 37b | CAGTTATACATGGTCAGCCC |
| 19 | 50,46 | CAGTTATACATGGTCAGCCC |
| 20 | 12g | TCCAGAGTCAGCCTCTTATG |
| 21 | 9 | GCCAATAGCCGGTCAGCCCT |
| 22 | 3c | CGCACGATGTCAGCCCTCG |
| 23 | 11 | GCCATACCGTGTCAGCCCCG |
| 24 | 18b | CCCAGTCAACCAATTGCTAG |
| 25 | 31c | CCAACCATCACCCGGTCCGG |
| 26 | 14 | TAACAAAGTGCCGGTCTGCC |
| 27 | 21 | GCCAGTCCAACCAATCCACG |
| 28 | 17,51,38 | CCGGTCGCTATACTGGGTCC |
| 29 | 16,41 | CGCGCCCACACCGTTTCCTG |
| 30 | 4 | TAGGCGCTGTTCCGTTAGGG |
| 31 | 59 | GAATCACATGACTGGTCGGA |
| 32 | 54 | GGGAGGGTTCTCCCCGTACG |
| 33 | 52,23 | CGCGGGGTGTCCGGCCGGCA |
| 34 | 45 | GTAGTGCGCTGAGCCGGGGA |
| 35 | 43 | GCACACGGTTCCCAATCATG |
| 36 | 39 | CCCAGACCGAAGCCAATCCC |
| 37 | 30a | GCCAGCCAAGACAACGCCCC |
| 38 | 30b | CCTGGTGCGGTGCGGGTGTC |
| 39 | 1a | GCTGATGAAGCCTCCGTCG |
| 40 | 15b | GAGGGGGCCACGGCAGTCCG |
| 41 | 3a | CAATACCCGTCCGTTTCCCC |
| 42 | 3b | CCACAACTCCTCAGCGGGTA |
| 43 | 7 | CGGTGGAGTCCTCCTGGTGC |
| 44 | 12c | CTCCACAGGGCCCCGTCTAG |
| 45 | 12e | GCACTATCCCCGCCCCACTC |
| 46 | 12f | AACACTGTGAACAGGCGTGC |
| 47 | 13b, 26b | CGCCCCAGTTCGCCAGTCGG |
| 48 | 15a | CACAAGCCCGTCATGTGTGG |
| 49 | 15c | CAGACAGCACTCAGCGCGCA |
| 50 | 19a | CATGAATTCTCCTATACT |
| 51 | 19b | ATCTGCACTATCCCGTCC |
| 52 | 25a | CCCTCCACCGATGGTAACCT |
| 53 | 31a | CCCGAAACCCACCACCCTTG |
| 54 | 31b | GTCGGCCCATCCTTTTCCA |
| 55 | 16a | CAAGGGCTGACCTTGCCGGG |
| 56 | 16b | CGCCCCAGTTCGCCAGTCGG |
| 57 | 1c | CACATACTGGACGGCTGACG |
| 58 | 5 | CCGCCATCTTCTCCTGAGC |
| 59 | 13a, 26a | CAAGGGCTGACCTTGCCGGG |
