# Supplementary material for: Interaction of the Chromatin Remodeling Protein hINO80 with DNA
Source: PLoS One. 2016 Jul 18;11(7):e0159370. doi: 10.1371/journal.pone.0159370 (PMC4948845; doi:10.1371/journal.pone.0159370)
Supplement: S4 Table — The numbers of putative sites of interaction of INO80 and YY1 proteins in the regions upstream of protein coding genes were analyzed. The sequences of YY1 motif we used for analysis are: 5’GCCATCAT3’ (8mer) and 5’CCGCCATNTT3’ (10mer) (www.genecards.org). (DOCX) [file pone.0159370.s009.docx]

| **Occurrence of binding motif** | | **Random sequence** |
| --- | --- | --- |
| INO80 protein | YY1 protein | 1126(8mer) |
| 4,696 (7mer) | 1300(8mer) |  |
| 334 (11mer) | 382(10mer) | 36(11mer) |

Supplementary Table 4. The occurrence of INO80 and YY1 protein binding sequence motif in the human genome

The numbers of putative sites of interaction of INO80 and YY1 proteins in the regions upstream of protein coding genes were analyzed. The sequences of YY1 motif we used for analysis are: 5’GCCATCAT3’ (8mer) and 5’CCGCCATNTT3’ (10mer) (www.genecards.org).
