# Supplementary material for: Interaction of the Chromatin Remodeling Protein hINO80 with DNA
Source: PLoS One. 2016 Jul 18;11(7):e0159370. doi: 10.1371/journal.pone.0159370 (PMC4948845; doi:10.1371/journal.pone.0159370)
Supplement: S5 Table — (DOC) [file pone.0159370.s010.doc]

Supplementary Table 5: List of putative INO80 targets in experimentally validated PRC1-PRC2 targets in mammals.
